# Functional Disruption of the Tomato Putative Ortholog of *HAWAIIAN SKIRT* Results in Facultative Parthenocarpy, Reduced Fertility and Leaf Morphological Defects

**DOI:** 10.3389/fpls.2019.01234

**Published:** 2019-10-14

**Authors:** Farida Damayanti, Fabien Lombardo, Jun-ichiro Masuda, Yoshihito Shinozaki, Takuji Ichino, Ken Hoshikawa, Yoshihiro Okabe, Ning Wang, Naoya Fukuda, Tohru Ariizumi, Hiroshi Ezura

**Affiliations:** ^1^Graduate School of Life and Environmental Sciences, University of Tsukuba, Tsukuba, Japan; ^2^Faculty of Life and Environmental Sciences, University of Tsukuba, Tsukuba, Japan; ^3^Faculty of Agriculture, University of Miyazaki, Miyazaki, Japan; ^4^Tsukuba Plant Innovation Research Center, University of Tsukuba, Tsukuba, Japan; ^5^Research Institute for Sustainable Humanosphere, Kyoto University, Kyoto, Japan; ^6^Biological Resources and Post-harvest Division, Japan International Research Center for Agricultural Sciences (JIRCAS), Tsukuba, Japan; ^7^Innovation Center, Nippon Flour Mills Co., Ltd, Atsugi, Japan

**Keywords:** Parthenocarpy, F-box gene, Tomato, miRNA, *HAWAIIAN SKIRT*, pollen development

## Abstract

A number of plant microRNAs have been demonstrated to regulate developmental processes by integrating internal and environmental cues. Recently, the *Arabidopsis thaliana* F-box protein *HAWAIIAN SKIRT* (*HWS*) gene has been described for its role in miRNA biogenesis. We have isolated in a forward genetic screen a tomato (*Solanum lycopersicum*) line mutated in the putative ortholog of *HWS*. We show that the tomato *hws-1* mutant exhibits reduction in leaflet serration, leaflet fusion, some degree of floral organ fusion, and alteration in miRNA levels, similarly to the original *A. thaliana hws-1* mutant. We also describe novel phenotypes for *hws* such as facultative parthenocarpy, reduction in fertility and flowering delay. In *slhws-1*, the parthenocarpy trait is influenced by temperature, with higher parthenocarpy rate in warmer environmental conditions. Conversely, *slhws-1* is able to produce seeds when grown in cooler environment. We show that the reduction in seed production in the mutant is mainly due to a defective male function and that the levels of several miRNAs are increased, in accordance with previous *HWS* studies, accounting for the abnormal leaf and floral phenotypes as well as the altered flowering and fruit development processes. This is the first study of *HWS* in fleshy fruit plant, providing new insights in the function of this gene in fruit development.

## Introduction

Plants tightly regulate their reproductive program based on internal and environmental cues to maximize reproduction success. A number of microRNAs (miRNAs) have been described for their role in regulating plant developmental processes by integrating physiological and environmental cues (D’Ario et al., 2017; Song et al., 2019). For example, miR156 levels are determined by internal factors such as plant age and sugar content, as well as external cues such as temperature and carbon dioxide concentration (Wang et al., 2009; May et al., 2013; Yang et al., 2013b). miR156 targets for degradation members of the *SQUAMOSA PROMOTER BINDING PROTEIN LIKE* (*SPL*) transcription factor family (Wu and Poethig, 2006). Some *SPL* members promote flower initiation by driving the expression of another miRNA, miR172, which targets *TARGET OF EAT* (*TOE*) floral initiation repressors (Jung et al., 2014). Thus, when favorable physiological and environmental conditions are met, decrease in miR156 and sequential increase in miR172 levels trigger the transition from the vegetative to the reproductive stage (Wu et al., 2009; Teotia and Tang, 2015).

Fruit set is triggered by pollination and fertilization (Gillaspy et al., 1993) and so fruit development, which is costly for the plant in resources, is coordinated with seed formation. Parthenocarpy is defined as fruit set without pollination and/or fertilization (Spena and Rotino, 2001; Martinelli et al., 2009). Fruit development is driven by hormonal changes in the ovary, mainly gibberellin, auxin, and cytokinin (reviewed in de Jong et al., 2009; Kumar et al., 2014). It has been shown that external application of any of these hormones can induce fruit development independently of fertilization, resulting in parthenocarpic fruits (Serrani et al., 2007a; Serrani et al., 2007b; Ding et al., 2013). Gibberellin levels regulate fruit development by modulating the activity of *GAMYB* transcription factors via the DELLA repressors (Achard et al., 2004). *GAMYB* messenger RNAs are also cleaved upon recognition by miR159 and overexpression of this miRNA results in anther development defects and reduction in fertility in *Arabidopsis thaliana* (Achard et al., 2004). The regulation of GAMYB levels by miR159 has also been demonstrated in tomato and overexpressor lines of miR159 show parthenocarpic fruits (da Silva et al., 2017). In barley however, it is the overexpression of *GAMYB* that results in a low-fertility phenotype, indicating species-specific responses to gibberellin in fruit development (Murray et al., 2003).

Obligate parthenocarpy is distinguished from facultative parthenocarpy. In the latter, seeds are formed when the sexual function is restored. It is the case for the tomato *parthenocarpic fruit* (*pat*) mutant, in which the severity of the floral defects is also function of the environmental conditions. When grown in favorable conditions, the *pat* mutant is able to produce seed-bearing fruits (Mazzucato et al., 1999). Adverse temperatures have been reported to affect negatively sexual organ development in tomato, with the male reproductive organ being most particularly sensitive to heat stress (Peet et al., 1998; Adams et al., 2001). As stamen development represses ovary development by maintaining low levels of gibberellin, a defective stamen development may induce parthenocarpy from premature gibberellin action (Medina et al., 2013; Okabe et al., 2018).

In *A. thaliana*, *HAWAIIAN SKIRT* (*HWS*) encodes an F-box protein and has been described as a new player in miRNA biogenesis and activity (Zhang et al., 2017; Lang et al., 2018). F-box proteins have been documented for their function within Skp1-Rbx1-Cul1-F-box protein (SCF) complexes in recruiting target proteins for degradation via the Ubiquitin 26S proteasome pathway (Hershko and Ciechanover, 1998; Gagne et al., 2002; Xu et al., 2009). The loss-of-function *athws-1* mutant shows global accumulation of miRNA levels (Lang et al., 2018). It has been hypothesized that HWS degrades non-optimal RNA-induced silencing complexes (RISCs; Mei et al., 2019). Other studies have suggested that *HWS* could be involved in early stages of miRNA biogenesis (Zhang et al., 2017; Lang et al., 2018). Alteration of miRNA levels in *athws-1* results in delay of floral organ abscission, reduction in leaf serration, floral organ fusion and cauline leaf fusion (González-Carranza et al., 2007; Lang et al., 2018). The leaf fusion and reduced serration phenotypes have been hypothesized to result from the degradation of the *CUP-SHAPED COTYLEDON1* (*CUC1*) and *CUC2* mRNAs via abnormal accumulation of miR164 (González-Carranza et al., 2017; Zhang et al., 2017). In addition, *HWS* has also been shown to be involved in root meristem activity in *A. thaliana* (Kim et al., 2016). Orthologs of *AtHWS* in rice and poplar have been reported as the *ERECT PANICLE 3* (*EP3*) and *PtaHWS* genes, respectively. In rice, the *ep3* mutant shows an erect panicle phenotype and reduced photosynthetic capacity (Yu et al., 2015). In poplar, accumulation of miR164e was observed in the mutant and a role for *PtaHWS* in root development under low nitrogen has been described (Dash et al., 2015).

To date, there is no description of the role of *HWS* in a fleshy fruit plant. In this study, we have isolated a tomato mutant line that shows facultative parthenocarpy, leaflet fusion, and reduction in leaflet serration. A mutation in the *Solyc01g095370* gene, the tomato putative ortholog of *AtHWS*, was found to be responsible for the mutant phenotype. The novel data contained in this study bring new insights in the function of *HWS* and particularly in its role in fruit development.

## Materials and Methods

### Plant Materials and Growth Conditions

The tomato (*Solanum lycopersicum*) mutants (TOMJPE8986, TOMJPW283, and TOMJPW3299) used in this study were isolated from an ethyl methanesulfonate (EMS) mutagenized population in cv. Micro-Tom genetic background (Saito et al., 2011; Shikata et al., 2016). M_5_ and M_6_ generations of TOMJPE8986 and TOMJPW283, and wild-type (WT) were used as plant material in parthenocarpy studies. For all other experiments, BC_3_ populations were used.

Near isogenic lines (NILs) in cv. Aichi First (beef-type tomato) and cv. Ueleie 106 WP (cherry tomato) genetic backgrounds were developed by crossing TOMJPE8986 (as male parent) to both cultivars (as female parents) and then backcrossed twice.

For parthenocarpy evaluation studies, characterization of backcross populations and allelism tests, plants were grown in greenhouses of Tsukuba Plant Innovation Research Center (T-PIRC) at the University of Tsukuba, Japan, in standard cultivation conditions as established in the University of Tsukuba. Plants were cultivated in greenhouses during the summer and autumn of 2016 and spring and autumn of 2017. Temperature and humidity values have been recorded hourly. Daily averages, daily minimum and daily maximum are presented in **Supplementary Figure S1**. All the plants were supplied with 1,000-fold-diluted Hyponex nutrient solution (Hyponex Japan Co., Ltd). For histological analysis, pollen viability measurement, Scanning Electron Microscope (SEM) observation and Real Time Quantitative PCR (RT-qPCR) analysis, plants were grown using 5 cm × 5 cm × 5 cm Rockwool cube (Grodan, Netherland) in a controlled-condition room as described in Okabe et al. (2018) and supplied with a commercial nutrient solution (Otsuka-A nutrient solution) (OAT Agrio Co., Ltd., Osaka, Japan).

For RT-qPCR of *SlHWS* on leaf tissues, WT and TOMJPE8986 seedlings were grown on a half strength Murashige and Skoog (MS) medium containing 0.8% agar. Seedlings were grown for 10 days in a controlled-condition room as described earlier and RNA was extracted as described hereafter.

### Gene Mapping

For the infinium SNP genotyping assay, an F_2_ population was generated from a cross between *Solanum pimpinellifolium* accession LA1589 and TOMJPE8986. Twenty-two F_2_ plants which exhibited fused leaflets were used and were genotyped for rough mapping using SolCAP’s Illumina Bead Chips (http://solcap.msu.edu/index.shtml) with 7,720 single nucleotide polymorphisms (SNPs) that were developed by SolCAP project. A total of 3,564 SNPs, which the physical position has been previously determined (Sim et al., 2012), were polymorphic between *S. pimpinellifolium* and TOMJPE8986. These SNPs were analyzed in the mapping population.

The genomic DNA from 29 F_2_ plants from a TOMJPE8986 × WT cross which showed fused leaflets was bulked. Whole genome sequence of the bulked DNA was obtained using an Illumina HiSeq 2000 next-generation sequencing platform. Bioinformatic analysis was performed and variants were called against tomato reference sequences of cv. Heinz 1706 (version SL2.40) and cv. Micro-Tom Japan as described previously (Ariizumi et al., 2014; Kobayashi et al., 2014).

### Genomic DNA Extraction

Genomic DNAs for both genotyping and whole genomic sequencing were extracted from leaves using a Maxwell 16 Tissue DNA Purification kits according to the manufacturer’s protocol (Promega, Madison, USA).

### Linkage Analysis Using Dcaps Marker

A dCAPS primer was designed using dCAPS finder 2.0 (Neff et al., 1998; **Supplementary Table S1**) and used to genotype six F_1_ plants and 128 F_2_ plants from a TOMJPE8986 × WT cross. The PCR program for dCAPS marker analysis consisted of an initial denaturation step for 2 min at 95^o^C, followed by 35 cycles of 30 s at 95^o^C, 20 s at 56^o^C, and 30 s at 72^o^C, followed by final extension for 5 min at 72^o^C, and incubation at 4^o^C until analysis. Each product was digested with *Nco*I at 37^o^C for 3 h. After digestion, each restriction-digested PCR products were subjected to electrophoresis and visualized in 3% (w/v) agarose gels in TAE.

### Tilling

The 9,216 EMS-mutagenized lines were screened to identify additional alleles of TOMJPE8986. The screening was performed by TILLING using a LI-COR DNA analyzer according to the procedure described by Okabe et al. (2011). A 1,831 bp region was amplified by PCR using fluorescent DY-681- and DY-781-labelled primers (**Supplementary Table S1**).

### Allelism Test

The F_1_ population for the allelism test was developed by crossing the homozygous line of TOMJPE8986 with both the homozygous line of TOMJPW283 and the heterozygous line of TOMJPW3299. Heterozygous line of TOMJPW3299 was used in the population development instead of homozygous line due to the difficulty to obtain seed from the homozygous TOMJPW3299 plant. A total of ten plants for each original mutant line and the WT were cultivated as control. Eight F_1_ plants from a TOMJPE8986 × TOMJPW283 cross and seven F_1_ plants from a TOMJPE8986 × TOMJPW3299 cross were scored for leaflet fusion and reduction in serration.

### Evaluation of Parthenocarpy and Seed Production

Evaluation of parthenocarpy trait was conducted in summer 2016, autumn 2016, and spring 2017. TOMJPE8986 was evaluated in all three different conditions, while TOMJPW283 was evaluated in summer and spring evaluations due to high percentage of cup-shape-like leaf and arrested apical meristem (AAM) phenotypes (**Supplementary Figure S2A**). At least 20 plants of each WT and homozygous TOMJPE8986 were grown in each evaluation, except for homozygous TOMJPW283 for which a minimum of 10 plants were grown in both evaluations. Throughout the study, “parthenocarpic fruits” refers to fruits developed from emasculated flowers only. In the case of non-emasculated or mechanically pollinated flowers, the resulting fruits are referred to as “seeded” or “seedless” whether seeds were observed. Flower emasculation was performed two days before anthesis to prevent self-pollination (Carrera et al., 2012). The number of emasculated flowers varied among cultivation seasons and depended on flower availability in each line evaluated. Percentage of parthenocarpy fruit formation was counted as the number of parthenocarpic fruit divided by the number of emasculated flowers. Mechanically pollinated flowers were also observed in both lines as control. Pollination was performed using an electric pollinator and carried out once a day. Parthenocarpic fruit formation and seedless-fruit formation was counted only from fruits at least 2 g of fresh weight so fruitlets were not included in the evaluation.

For evaluation of the sexual function and seed production experiments, TOMJPE8986 (*slhws-1*) was crossed to the WT and plants were grown in a greenhouse in autumn 2017 as described earlier. Flowers were emasculated two days before anthesis then hand-pollinated at anthesis with pollen from a different flower in each WT × WT; WT × *slhws-1*; *slhws-1* × *slhws-1*, *slhws-1* × WT cross combination. The number of seeds was counted from 15 fruits for each cross combination.

Measurement of ovary diameter from emasculated and mechanically pollinated flowers were conducted in five time points (two days before anthesis, anthesis, two days after anthesis, four days after anthesis, and eight days after anthesis) to study the early fruit development in BC_3_ TOMJPE8986. All measurements were based on 15 ovaries per time point per line, which were randomly sampled from 25 individual plants per line.

### Pollen Quantification and Viability Observation

Pollen viability was determined in an Alexander staining (Alexander, 1969) with modification (Peterson et al., 2010) and confirmed in pollen germination experiments. Thirty-six flowers at anthesis stage of each WT and BC_3_ TOMJPE8986 line were collected randomly from 15 plants at three different days. Anther cones were placed in 1.5 ml tubes, and 100 µl of Alexander stain solution was added to each anther cone. Pollen grains were subsequently carefully released from the anther cones using a 1 µl pipette tip and samples were incubated at 37^o^C for 3 h. Pollen grain observation and counting were performed using a cell counter. The number of pollen grains was counted and determined according to the manufacturer’s protocol (Biomedical Science Co., Ltd., Tokyo, Japan).

For *in vitro* pollen germination, pollen grains from 25 flowers at anthesis stage for each line were collected in 1.5 ml tube and spread onto germination medium (Hicks et al., 2004) using a small brush. Pollen grain was considered as germinated pollen if the length of pollen tube was at least two times its diameter. For *in vivo* pollen germination, each flower was self-pollinated and covered by a small paper bag to prevent cross-pollination. After 24 h, pollinated pistils were collected and treated as previously described by Yu and Parthasarathy (2014) with some modification. All the microscopic pollen grain observation was carried out under an Olympus BX53 light microscope (Olympus Corporation, Tokyo, Japan) using an imaging software (cellSens Standard 1.6, Olympus, http://www.olympus-global.com). In the case of *in vivo* pollen germination, UV light was used as light source instead of the standard light source from the microscope. At least three biological samples were observed in each BC_3_ TOMJPE8986 and the WT line.

### Characterization of TOMJPE8986 Backcross Population

Characterization of the mutant was carried out during the autumn cultivation. Twenty plants of WT and 14 BC_3_ plants of TOMJPE8986 were grown in autumn 2017 and observed for plant-related traits. Data of fruit quality-related traits were collected from 50 fruits per line, which were sampled randomly, except for fruit color and fruit firmness which were measured on 20 fruits per line per measurement. All the fruits were analyzed at breaker stage (Br)+10 days.

Total soluble solid (TSS) was measured in individual fruits using a refractometer PAL-J (Atago Co., Ltd., Tokyo, Japan). The TSS was measured in triplicates from the pericarp tissue after removing seeds and the fruit jelly part. Fruit color was analyzed using a Minolta Color Reader CR-10 (Konica Minolta Sensing, Inc., Osaka, Japan) based on the Commission Internationale de l’Eclairage Laboratory (CIELAB) color space analysis. Color was measured at two different random points in the equatorial part of each fruit. Three color parameters were represented as the lightness/brightness of the color (L*), the red to green axis (a*), and the yellow to blue axis (b*). Lycopene content was estimated from the a*/b* ratio as reported by Arias et al. (2000). Fruit firmness was measured with a CT3 texture analyzer (AMETEK Brookfield, Middleboro, USA) set at a 1 mm/s speed to an 8 mm depth.

### Histological Analysis

Histological analysis was carried out on flower buds of both BC_3_ TOMJPE8986 and the WT at stage 9 and 16 (3 and 8 mm respectively; Brukhin et al., 2003), which were collected randomly from 25 plants for each line, according to Chusreeaeom et al. (2014) with the following modifications. Bud and flower sections were stained with 0.05% Toluidine blue after deparaffinization with xylene. The sections were observed under an Olympus BX53 light microscope (Olympus Corporation, Tokyo, Japan) using an imaging software (cellSens Standard 1.6, Olympus, http://www.olympus-global.com). At least three biological samples were observed for each stage in each line.

### Scanning Electron Microscope (SEM) Observation

Twenty flowers at anthesis stage were sampled from 25 plants of each BC_3_ TOMJPE8986 and WT line. All organs but the anthers were carefully removed. Anther cones were then gently opened before observation under a Hitachi Tabletop Microscope TM3000 (Hitachi, Tokyo, Japan). Each sample was captured in three different magnification (40, 80, and 120×). Anther dehiscence was evaluated based on pollen sac opening size and pollen grain dispersion in each sample observed at 80× magnification.

### Real Time Quantitative PCR (RT-qPCR) Analysis

Leaves were collected from 10-day-old seedlings and separated into three biological replicates for each WT and BC_3_ TOMJPE8986 line. Each biological replicate consisted in leaves sampled from seven individual plants. Buds at stage 9 (corresponding to 3 mm-long buds as defined in Brukhin et al., 2003) were collected and separated into three biological replicates for each WT and BC_3_ TOMJPE8986 line. Each biological replicate consisted in a final 10-bud sample using two buds from five plants. Plants were selected based on their flowering stage from 20- and 40-individual populations for the WT and BC_3_ TOMJPE8986 lines, respectively. High Pure miRNA Isolation Kit (Roche, Mannheim, Germany) was used to extract total RNA from buds at stage 9 samples. RNA purification was performed by “RNA Clean and Concentrator 5” (Zymo Research, California, USA). The cDNA was synthesized from 1 mg of total RNA using a Superscript IV VILO Master Mix with ezDNase enzyme (Life Technologies, California, USA). Two-tail based RT-qPCR primers were used in cDNA synthesis for miRNA RT-qPCR analysis (**Supplementary Table S1**). Synthesized cDNA was diluted to 10-folds to be used for RT-qPCR experiments. The RT-qPCR was performed using the C1,000 Thermal cycler CFX96 Real-Time System (BIO-RAD, California, USA). The reaction was performed in a 25 ml volume as follows: 95^o^C for 30 s for initial denaturation, followed by 40 cycles of 5 s at 95^o^C, 30 s at 60^o^C, 10 s at 95^o^C, and 5 s at 65^o^C. The expression level of each gene was normalized to the expression of SAND (*Solyc03g115810*), which was used as an internal control. The primer sequences used in the experiment are shown in **Supplementary Table S1**. Primer design and RT-qPCR of the miRNAs was carried out as described by Androvic et al. (2017).

## Results

### Identification of an F-Box Gene Involved in Parthenocarpic Fruit Development

**Figure 1:**
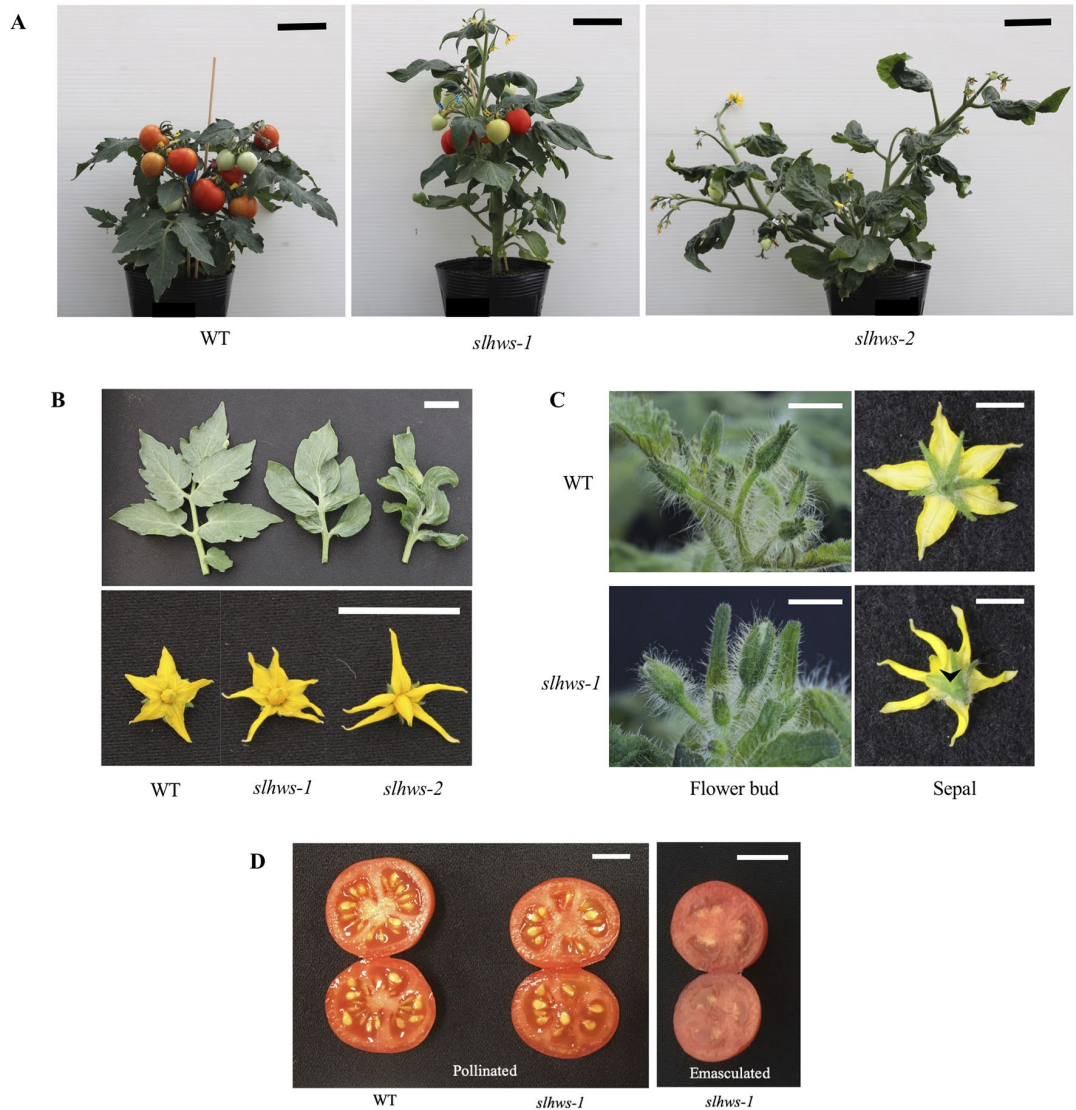
Phenotypes of *slhws-1* (TOMJPE8986) and *slhws-2* (TOMJPW283). **(A)** Overall plant architecture in *slhws-1* and *slhws-2*. Scale bars = 5 cm. **(B)** Fused leaflet and reduction in leaflet serration (top) and flower phenotypes (bottom) in *slhws-1* and *slhws-2*. Scale bars = 2 cm. **(C)** Bud and flower phenotypes in *slhws-1*. Arrow indicates fused sepals. Scale bars = 1 cm. **(D)** Fruit phenotypes from mechanically pollinated and emasculated flowers in *slhws-1*. Scale bars = 1 cm.

An EMS-mutagenized tomato population of *S. lycopersicum* cv. Micro-Tom has been generated in an earlier study (Saito et al., 2011). In order to identify genes involved in parthenocarpic fruit development, the population was screened for seedless fruits and the line TOMJPE8986 was isolated. In addition to the seedless-fruit trait, increased plant height, fused leaflets and reduction in leaf serration were also striking features of TOMJPE8986 traits (**Figures 1A, B**). The fused-leaflet trait was used in further work to distinguish the mutant from the WT as it can easily be scored visually. Preliminary genetic analysis in a backcross F_2_ segregant population showed a distribution of wild-type (WT) and mutant phenotypes fitting a 3:1 ratio (χ^2^ = 0.53, *p* > 0.47; **Supplementary Table S2**), typical of an EMS-induced monogenic recessive mutation.

**Figure 2:**
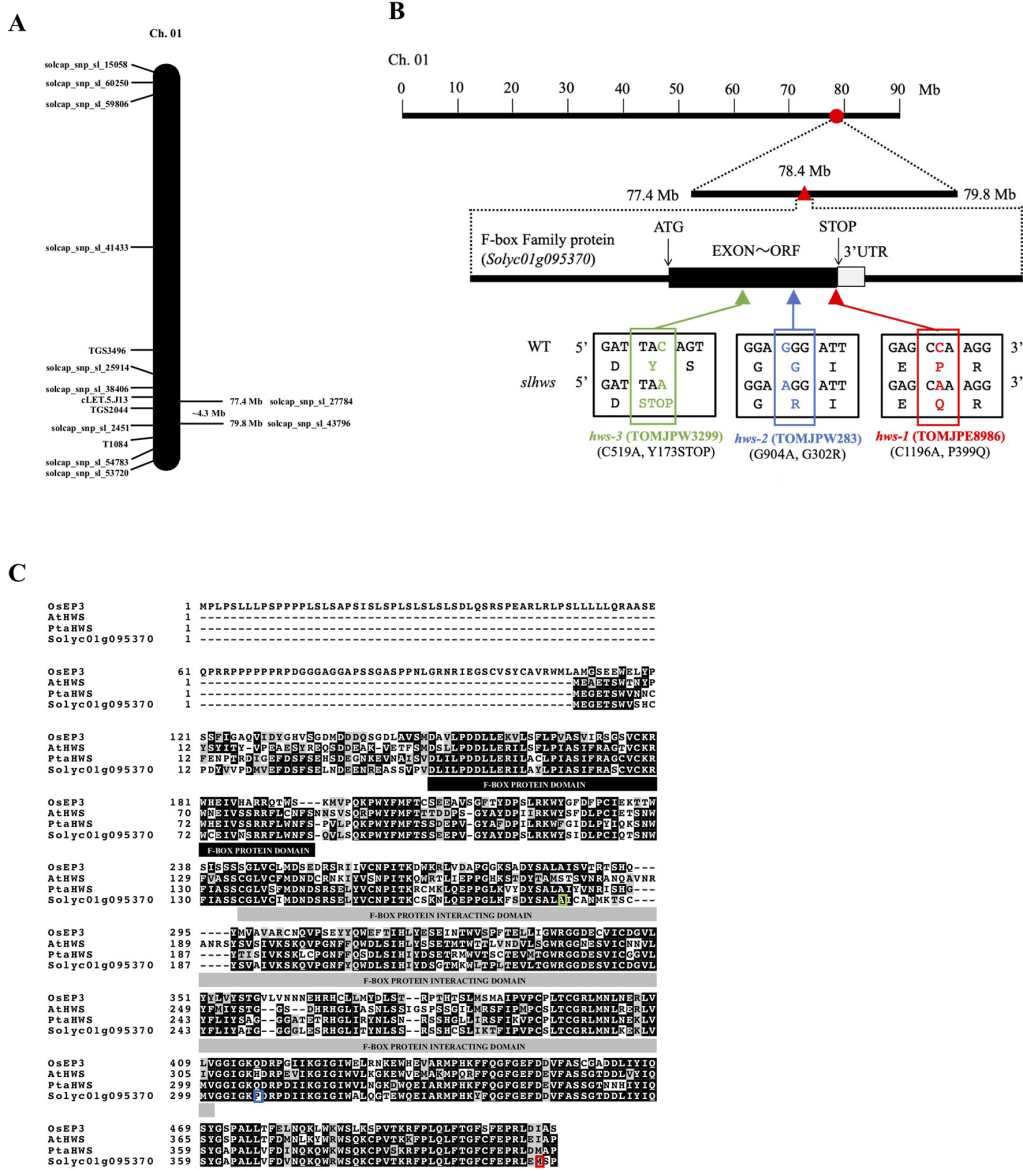
Genetic mapping of the *slhws-1* mutant. **(A)** Rough mapping of chromosome 1 using SolCap array. **(B)** Location and gene structure of *Solyc01g095370*. Red circle indicates the candidate area determined from the SolCap array. Red triangle indicates the mutation location identified by whole genome sequence. The black and grey boxes indicate exon and 3’UTR, respectively. Mutation details and resulting amino acid changes are indicated for all *slhws* alleles. **(C)** Sequence alignment of proteins encoded by *Solyc01g095370* and other *HWS* orthologs.

To identify the causal gene of the TOMJPE8986 phenotype, an SNP infinium assay was performed using a cross between the mutant line and *S. pimpinellifolium* accession LA1589. A total of 3,564 SNPs was identified, and calculation of allele frequencies revealed linkage of the mutant phenotype to seven SNPs on chromosome 1 (**Supplementary Figure S3**). Using the SolCap array database (http://solcap.msu.edu), the candidate region for the causal gene in TOMJPE8986 was further delimited to a 2.4 mega base (Mb) interval, around the 77.4 Mb and 79.8 Mb positions (**Figures 2A, B**). Whole genome sequencing of both mutant and WT lines revealed a total of 187 homozygous mutations in the candidate region, consisting in 129 insertions and deletions (INDELs) and 58 point mutations (**Supplementary Table S3**). Among the INDELs, 116 are intergenic and 13 intragenic, of which 12 are within introns and one in a 5′ untranslated region. Among the 58 point mutations, 50 are located within intergenic regions, five within introns, one in a 3′ untranslated region and two within exons, one of which being silent. The remaining exonic mutation is a cytosine to adenosine transversion located towards the 3′ end of the coding sequence (CDS) of the *Solyc01g095370* gene, which is annotated as an F-box cyclin-like protein encoding gene. To confirm that the fused-leaflet phenotype co-segregates with the mutation in *Solyc01g095370*, a dCAPS marker was designed and an additional 128 F_2_ plants was scored. All homozygous plants for the mutation showed fused leaflets and the segregation ratio was not significantly different to the expected 1:2:1 (WT:heterozygous:mutant) for a monogenic recessive mutation (χ^2^ = 3.19, *p* > 0.2; **Supplementary Table S4**).

Analysis of the CDS of *Solyc01g095370* showed that the mutation results in a Proline to Glutamine amino acid change (P399Q) in the 405 amino acid-long protein (**Figure 2**). Blast searches against protein databases indicate that the protein encoded by *Solyc01g095370* contains an F-box domain (amino acids 43–86) and an F-box protein interaction domain (amino acids 135–300). Searching against the *A. thaliana* nucleotide collection identified *Solyc01g095370* as a putative ortholog of the *AtHWS* F-box gene. Alignment of the AtHWS and Solyc01g095370 proteins revealed more than 65 percent of amino acid identity (**Figure 2C**).

To isolate additional alleles of TOMJPE8986, our mutant collection was screened using the TILLING method. Two other lines were identified, TOMJPW283 and TOMJPW3299, carrying a missense mutation and a mutation resulting in a premature STOP codon, respectively (**Figure 2**). Both lines showed morphological leaf defects similar to the ones observed in TOMJPE8986, with TOMJPW283 showing an intermediate phenotype and TOMJPW3299 being the most severely affected (**Figures 1A, B**; **Supplementary Figure S4**). Allelism tests revealed that both TOMJPW283 and TOMJPW3299 lines were allelic to TOMJPE8986. All the progeny from a TOMJPE8986 × TOMJPW283 cross showed fused leaflets and reduction in leaflet serration similar to the ones observed in the parental lines (**Supplementary Figure S4**). Among seven F_1_ plants from a cross between homozygous TOMJPE8986 and heterozygous TOMJPW3299, three showed a WT-like leaf phenotype and four showed a mutant-like phenotype. This segregation ratio is not significantly different to the expected 1: 1 (heterozygous: mutant) ratio (χ^2^ = 0.14, *p* > 0.71; **Supplementary Table S5**).

The TOMJPE8986 was also crossed to two different cultivars, cv. Aichi First and cv. Ueleie 106WP, to generate two NILs BC_2_ populations. NILs individuals showed very similar leaf phenotypes to the ones observed in the original TOMJPE8986 mutant (**Supplementary Figure S5**).

Altogether, these results identify *Solyc01g095370* as the candidate causal gene for the mutant phenotype in TOMJPE8986. Alike the phenotype of TOMJPE8986, reduction in leaf serration and sepal and cauline leaf fusions have been shown in *athws* mutants (González-Carranza et al., 2007; Lang et al., 2018). Based on this observation as well as the high homology between AtHWS and TOMJPE8986 and additional data later described in this study, TOMJPE8986, TOMJPW283, and TOMJPW3299 are designated *slhws-1, -2* and -3 onwards, respectively. While *slhws-1* produced enough seeds to perform experiments conveniently, both *slhws-2* and *slhws-3* produce only few or almost no seeds, respectively. For this reason, most of the study is focused on the *slhws-1* allele.

### *Slhws* Mutants Show Facultative Parthenocarpy

To further evaluate the parthenocarpy trait and its stability in *slhws* mutants, plants were grown in different seasons and flowers were emasculated two days before anthesis. It has been reported that high temperatures promote parthenocarpic fruit formation in tomato (Sato et al., 2001). Several studies describe a day/night temperature cycle of 32 °C/26 °C to induce heat stress (HS) (Peet et al., 1998; Sato et al., 2001; Pressman et al., 2002; Paupière et al., 2017), which corresponds to a daily average of 29 °C. Consistently, Peet et al. (1998) have observed that fruit production decreases of about 90% when plants are grown with a daily average temperature of 29 °C. Days with an average of 29 °C or above were therefore defined as HS days and days with a maximum temperature of 32 °C or above as elevated maximum temperature (EMT) days. The optimal humidity conditions are generally thought be in the 50–70% range and extreme values (below 30 or above 90%) can cause pollen development abnormalities (Peet et al., 2002). During our autumn cultivation, only six EMT days and no HS days were recorded and the atmosphere was rather dry (**Table 1**; see also **Supplementary Figure S1** for full data). In these conditions, no parthenocarpic fruits were formed on WT plants (**Table 2**). In spring, conditions were similar than the ones in autumn, however it was more humid and the number of EMT days was more than doubled (**Table 1**). Very few parthenocarpic fruits (2%) developed from emasculated WT flowers. During summer, temperatures were high, with 15 HS days and 34 EMT days, and humidity conditions were relatively favorable (**Table 1**). Parthenocarpy was rarely observed in the WT, with 4% of parthenocarpic fruits counted (**Table 2**). In contrast to the WT, *slhws-1* showed markedly higher fruit formation rates in all three seasons, with 11, 41, and 59% in autumn, spring, and summer respectively (**Table 2**). Higher parthenocarpy rates were also observed for *slhws-2* in comparison to the WT, however not to the extent of *slhws-1*, most likely because the flowers of *slhws-2* were more fragile due to the stronger morphological defects.

**Table 1:** Number of days with heat and humidity-related stresses in the three seasons of cultivation.

Season	Temperature		Hiumidity	
	HS (day)	EMT (day)	RH_avg_ <50% (day)	RH_avg_ >70% (day)
Autumn	0	6	24	0
Spring	0	14	11	4
Summer	15	34	2	8

Heat stress (HT) days are defined as days with an average temperature of 29°C or above. Elevated maximum temperature (EMT) days are defined as days with a maximum temperature of 32°C or above. RH_avg_, daily mean relative humidity. The data correspond to the 40-day long flowering stage in each season.

**Table 2:** Evaluation of parthenocarpy trait in three seasons.

Line	Parthenocarpic fruit formation from emasculated flowers		
	Autumn	Spring	Summer
	Seedless fruit formation in mechanically pollinated flowers		
	Autumn	Spring	Summer
WT	0/200 (0%)	5/207 (2%)	25/593 (4%)
*slhws-1*	23/200 (11%)	47/116 (41%)	23/39 (59%)
*slhws-2*	n.d.	8/47 (17%)	7/62 (11%)
WT	0/50 (0%)	1/50 (2%)	4/50 (8%)
*slhws-1*	17/50 (34%)	46/50 (92%)	18/18 (100%)
*slhws-2*	n.d.	42/50 (84%)	15/15 (100%)

Only 2 g fruits were included in calculations of parthenocarpic fruit and seedless-fruit formation ratios. n.d., data was not determined.

**Figure 3:**
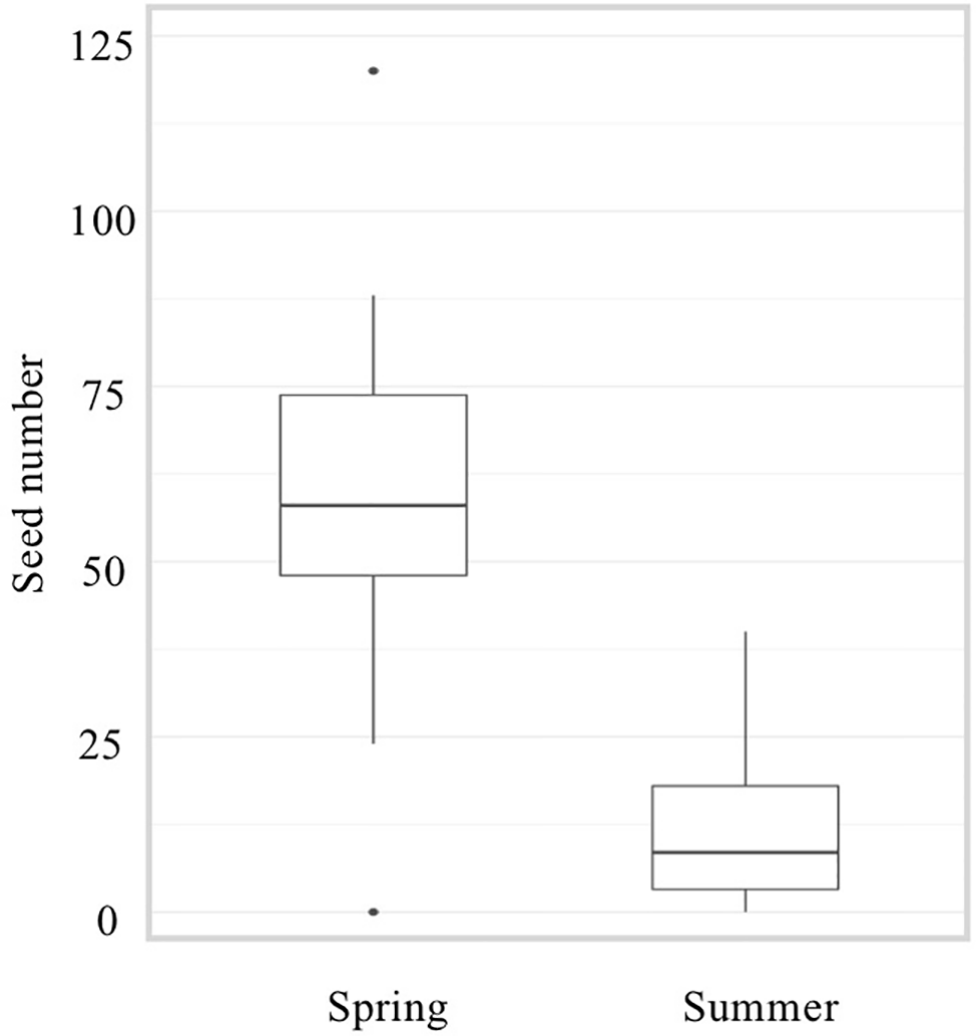
Number of seeds in the WT during spring and summer cultivations. Number of seeds per fruit was counted from 50 fruits derived from mechanically pollinated flowers.

Peet et al. (1998) have reported a dramatic reduction of about 80% in seed number when plants were grown under HS conditions. A similar decrease was observed when comparing seed number from our spring and summer cultivations (**Figure 3**). Observations of fruits derived from mechanically pollinated flowers of *slhws-1* and *slhws-2* revealed that seeds were occasionally produced, demonstrating that the mutants are facultative parthenocarpic (**Figure 1D**). In spring, both *slhws-1* and *slhws-2* produced a very low amount of seeds, with averages below 1 seed per fruit (0.1 and 0.3 seed, respectively; data not shown). Not a single seed could be obtained in the summer cultivation, with 100% of seedless fruits observed in this season (**Table 2**).

Altogether, these results revealed that parthenocarpic rate (emasculated flowers) and seed formation (mechanically pollinated flowers) were strongly influenced by HS in *slhws-1* and *slhws-2*.

**Figure 4:**
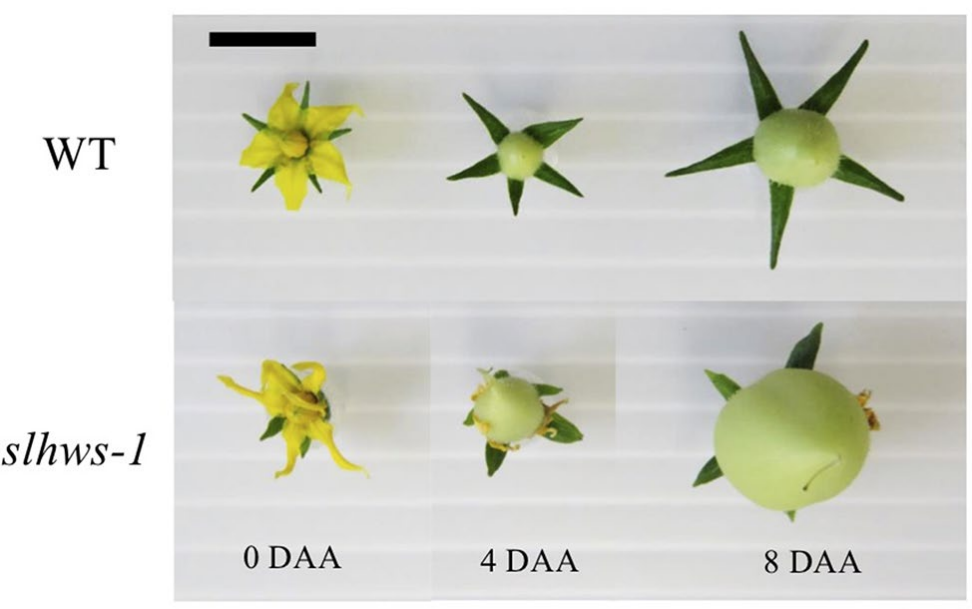
Ovary size of *slhws-1*. Ovary size was observed from mechanically pollinated flowers. Scale bar = 1 cm.

Ovary growth has been reported to be faster in some parthenocarpy mutants (Carrera et al., 2012; Shinozaki et al., 2015). In our experiments, ovaries developed from mechanically pollinated flowers of *slhws-1* were growing three times faster than their WT counterparts between 2 DAA and 4 DAA (**Table 3**; **Figure 4**). At 8 DAA, the ovary growth rate of the mutant was similar to the one of the WT, however the ovary of *slhws-1* was still significantly larger than the WT one at that stage. In the case of emasculated flowers, most of WT ovaries stopped growing after anthesis (0 DAA) and flower would drop off at around 8 DAA since pollination is necessary to trigger fruit development in the WT. In contrast, ovaries of the mutant continued to enlarge after anthesis, although the growth rate was lower than the one measured using mechanically pollinated flowers (**Table 3**).

**Table 3:** Ovary growth rate in *slhws-1*.

Stage(DAA)	Emasculated flowers				Mechanically pollinated flowers			
	WT		*slhws-1*		WT		*slhws-1*	
	Ovary diameter (mm)	Growth rate (mm/day)	Ovary diameter (mm)	Growth rate (mm/day)	Ovary diameter (mm)	Growth rate (mm/day)	Ovary diameter (mm)	Growth rate (mm/day)
-2	0.9 ± 0.0	–	1.1 ± 0.0*	–	0.9 ± 0.0	–	1.1 ± 0.0*	–
0	1.1 ± 0.0	0.1	1.3 ± 0.0*	0.1	1.0 ± 0.0	0.1	1.2 ± 0.0*	0.1
+2	1.1 ± 0.0	0.0	1.6 ± 0.1*	0.2	1.4 ± 0.1	0.2	1.6 ± 0.1*	0.2
+4	1.1 ± 0.1	0.0	2.7 ± 0.4*	0.5	2.5 ± 0.3	0.5	4.5 ± 0.3*	1.5
+8	n.a.	n.a.	7.0 ± 0.3 ^n.a.^	1.1	8.8 ± 0.5	1.6	10.8 ± 0.4*	1.6

n.a., data not applicable since at +8 DAA all emasculated WT flowers have died. Values are mean SE of 15 ovaries. *, significantly different from the mean value of wild type according to a t-student test P < 0.05.

### The Male Function Is Impaired in *Slhws-1*

#### *Slhws-1* Anthers Show Lower Dehiscence

**Figure 5:**
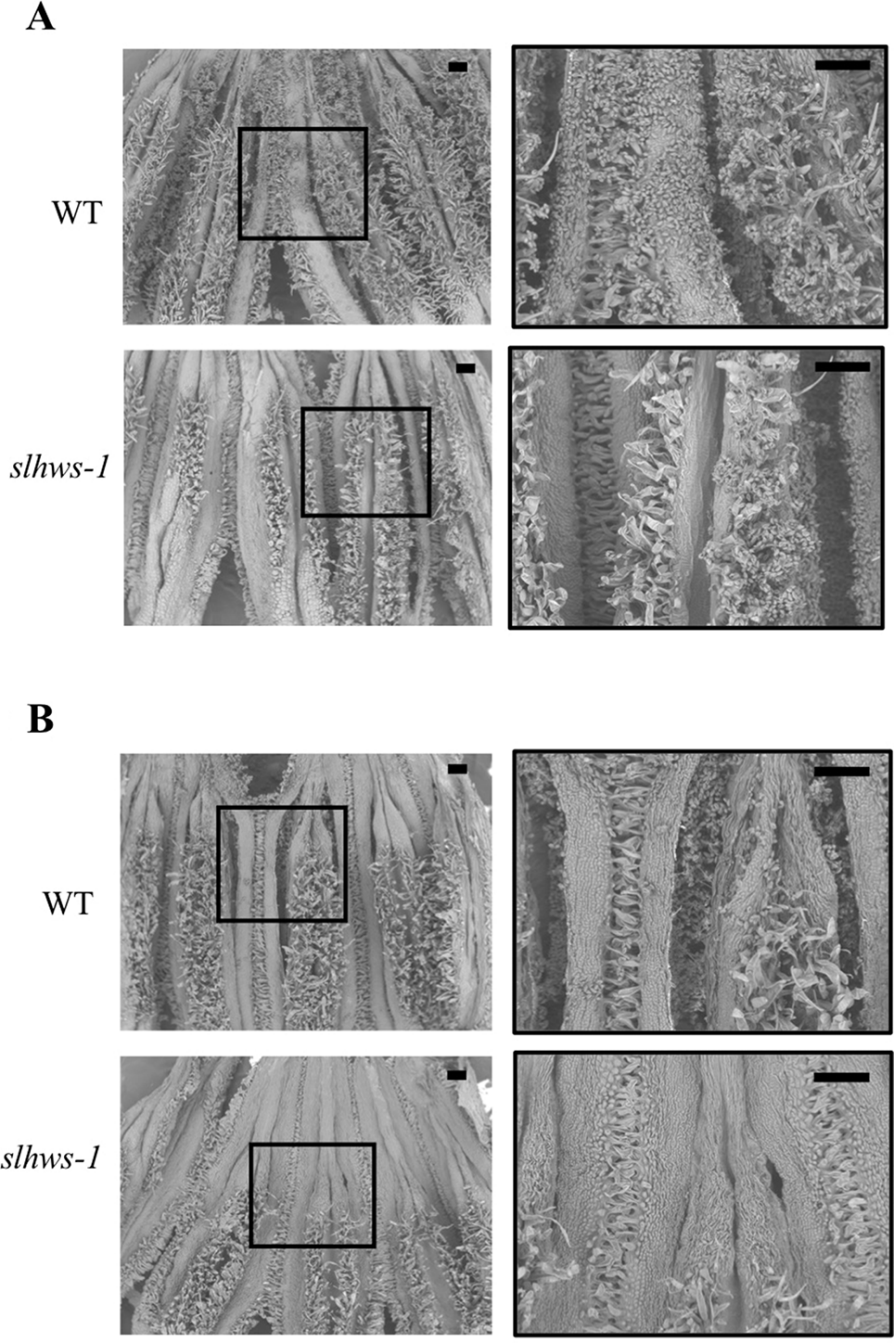
Scanning electron microscopy of *slhws-1* anthers at anthesis. **(A)** Representative anthers with high dehiscence in WT and *slhws-1*. **(B)** Representative anthers with low dehiscence in WT and *slhws-1*. Boxes indicate magnified areas. Scale bars = 1 mm.

**Figure 6:**
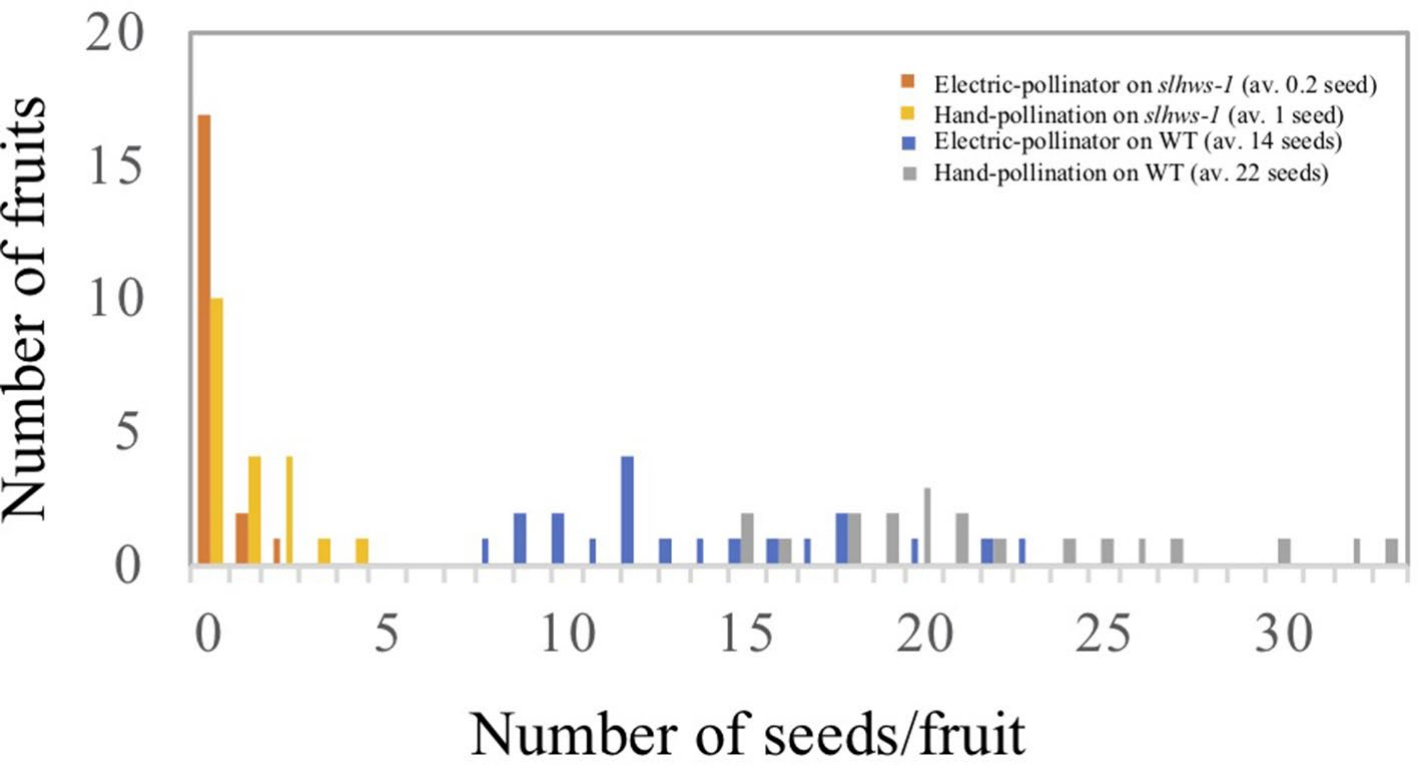
Number of seeds using two different pollination methods. Number of seeds per fruit was counted from 20 fruits in each WT and *slhws-1* line for each pollination method.

Heat stress is known to impair anther development, resulting in lower fruit set in tomato (Sato et al., 2001). Our previous experiment showed that *slhws-1* and *slhws-2* were unable to produce seeded fruits during the summer cultivation, when plants are subject to HS (**Tables 1** and **2**). To further investigate the male function in the mutant, plants were grown in a controlled-condition room and flowers at the anthesis stage were observed using SEM. Pollen sacs in the WT were widely open, while those of the mutants were only partly open, showing fewer pollen grains released (**Figure 5**). To confirm that a lower dehiscence was responsible for the poor seed production in the mutant, flowers were self-pollinated either by gently applying pollen onto the stigma by hand or mechanically by using an electric pollinator and seeds were counted. Hand-pollination almost doubled the number of seeds per fruit in the WT. In contrast, the average number of seeds in mutant fruits was only slightly increased by hand-pollination (**Figure 6**). This result indicates that the poor dehiscence observed in SEM experiment is not the only cause for the low seed production in *slhws-1*.

#### *Slhws-1* Produces Fewer and Less Viable Pollen Grains

**Figure 7:**
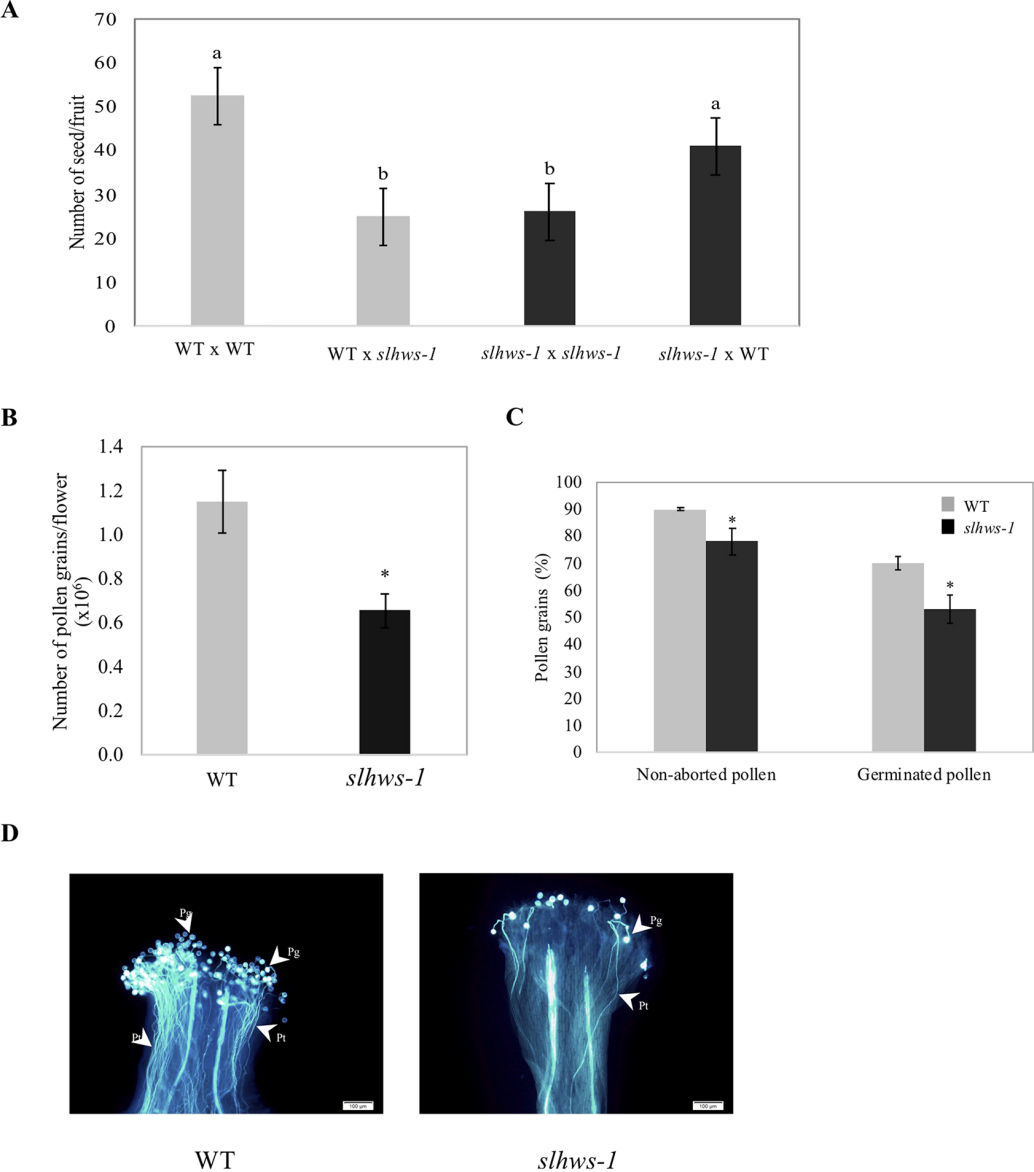
Evaluation of the sexual function of *slhws-1*. **(A)** Number of seeds per fruit obtained from different cross combinations. Grey bars correspond to crosses with the WT used as female parent. Black bars indicate crosses with *slhws-1* used as female parent. All flowers were emasculated two days before anthesis and then self-pollinated or cross pollinated. Values are mean SE of 15 fruits. Mean values with the same letter are not significantly different based on Tukey-Kramer test at *P* <0.05. **(B)** Pollen quantified using a cell counter after Alexander staining. Values are mean SE of 12 replicates, each replicate consisted of three flowers. *, significantly different from the mean value of the WT according to a t-student test *P* < 0.05. **(C)** Percentage of aborted pollen counted after Alexander staining. Values are mean SE of 12 replicates, each replicate consisted of three flowers. For percentage of *in vitro* germinated pollen, values are mean SE of three replicates. Percentage was calculated from >1000 pollen grains for each replicate. *, significantly different from the mean value of the WT according to a t-student test *P* < 0.05. **(D)** Pollen *in vivo* germination after staining with 0.01% aniline blue. *Pg*, pollen grain; *Pt*, pollen tube.

To determine which sexual organ function is affected in the mutant, seed production in fruits derived from reciprocal crosses between *slhws-1* and the WT was analyzed. Using mutant pollen on WT flowers resulted in a low seed number, indicating that the mutant pollen was defective. On the other hand, using WT pollen on mutant flowers resulted in a number of seeds only slightly lower than the WT control, indicating that poor pollen quality was the major determinant for the low seed production observed in the mutant (**Figure 7**). The amount of pollen produced by the mutant was quantified in a condition-controlled environment using a cell counter and it was found that the pollen grain number for *slhws-1* was roughly half of that of the WT (**Figure 7**). Pollen viability was also reduced in *slhws-1* as evidenced by the lower percentage of non-aborted pollen grains in an Alexander staining test and the lower germination rate observed in an *in-vitro* test (**Figure 7**). Still, no significant differences in size could be observed between WT and mutant non-aborted pollen grains (data not shown). The low number of pollen grains, the poor dehiscence and decreased pollen viability altogether resulted in an inefficient fertilization (**Figure 7**).

#### Defects in Stamen Development in *Slhws-1*

**Figure 8:**
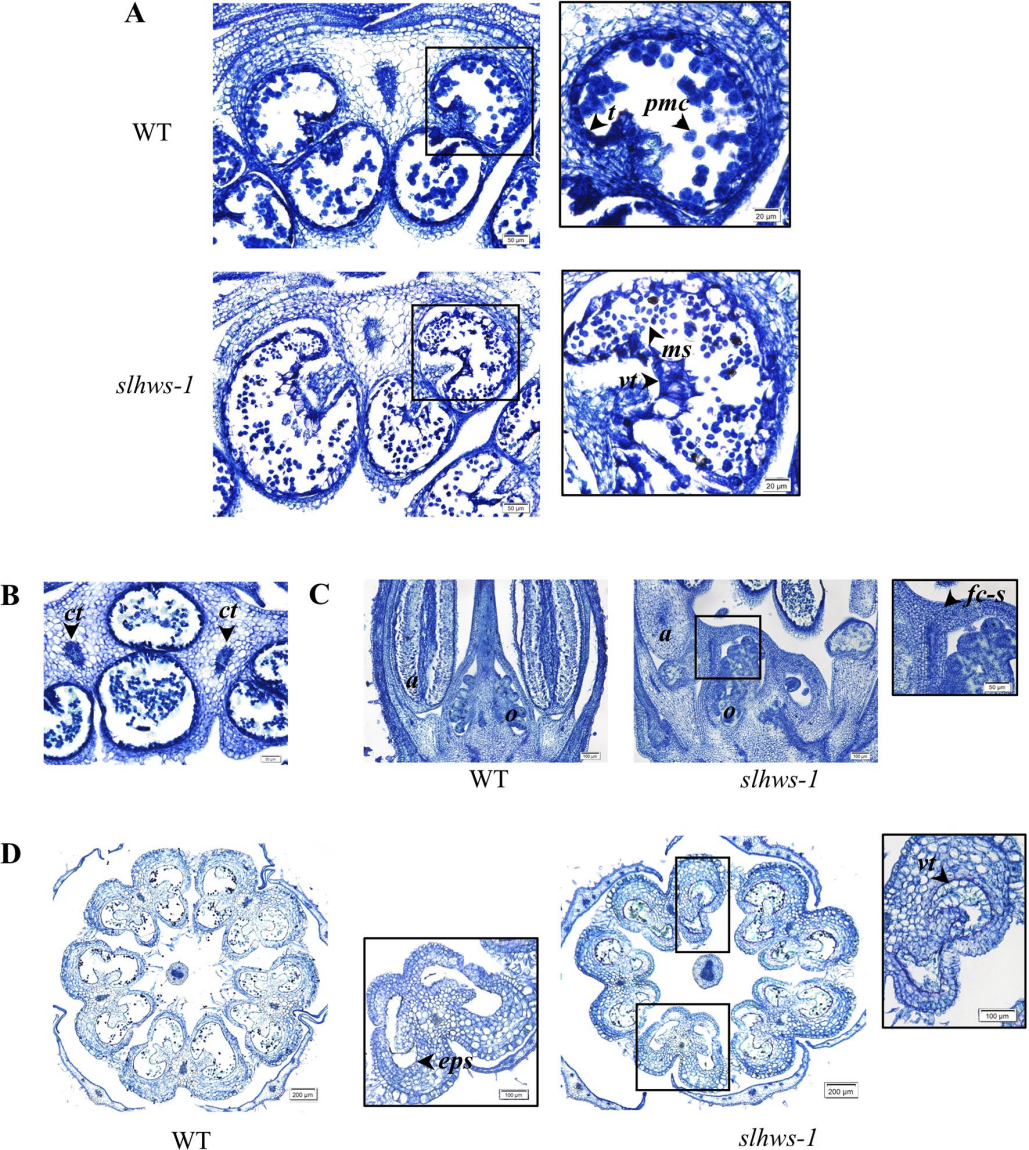
Histological analysis of flower buds. **(A)** Transversal sections of stage 9 flower buds. Tapetum (*t*), tetrads enclosed inside a pollen mother cell (*pmc*). Vacuolated tapetum (*vt*) and tetrads (*td*) released from *pmc*. **(B)** Transversal sections of fused pollen sacs of *slhws-1* stage 9 buds. *ct*, connective tissue. **(C)** Longitudinal sections of stage 9 buds. Arrow indicates carpel-to-stamen fusion (*fc-s*). *o*, ovule. *a*, anther. **(D)** Transversal sections of stage 16 flowers. *eps*, empty pollen sac characteristic of the mutant; *vt*, vacuolated tapetum.

Anther-stigma fusion was previously observed on about twenty-five percent of flowers during the parthenocarpy evaluation (data not shown). To further investigate the cause of the defective male function in the mutant, flower histological sections at the pollen mother cell (PMC), at meiosis stage (stage 9) and pollen at mitosis stage (stage 16) were observed under the microscope. Mutant flowers at stage 9 occasionally showed an abnormal tapetum as well as release of microspore from the tetrads, which should normally occur at stage 12 (Brukhin et al., 2003; **Figure 8**). Anther-carpel fusion was seen on approximately twenty-five percent of observed flowers and anther-anther fusion was seen at an estimated frequency of 10% (**Figures 8B, C**). In severe cases, the tapetum was vacuolated and pollen sacs were empty (**Figure 8**), indicating a defective microsporogenesis in *slhws-1*. These defects are comparable to the ones described in a number of male sterility mutants (Kim et al., 2010; Zhu et al., 2011; Jeong et al., 2014).

### Plant Architecture Is Altered in *Slhws* Mutants

Plant height in *slhws-1* was markedly increased when measured at 60 days after sowing, with plants about 9 cm taller on average (**Figure 1**; **Table 4**). Longer internodes contributed to the increased height and the fewer lateral shoots indicated that apical dominance was stronger in the *slhws-1* mutant (**Table 4**). Stem diameter was also significantly larger and the number of flowers of *slhws-1* was about a third lower. While *slhws-1* and *slhws-2* consistently showed fused-leaflet and reduction in leaflet serration phenotypes (**Figure 1**), their severity was largely influenced by the culture conditions. During summer cultivation, when temperatures are high, leaflet fusion could be seen as early as the fourth leaflet. In contrast, leaflet fusion occurred as late as the seventh leaflet when the growth conditions were more favorable (data not shown). Close examination of *slhws-1* flowers revealed barrel-shaped buds with slightly short sepals which were unable to cover completely the anther cone (**Figure 1C**). The number of sepals was also increased (**Table 4**) and sepal fusion was commonly observed from the second inflorescence (**Figure 1C**), reminiscent of the phenotype observed in the *athws-1* mutant (González-Carranza et al., 2007).

**Table 4:** Characterization of *slhws-1* in the autumn season.

Traits	WT	*slhws-1*
**General architecture**		
Plant height (cm)^a^	8.8 ± 0.2	8.0 ± 0.4
Plant height (cm)^b^	16.0 ± 0.5	25.2 ± 1.1*
Stem diameter (mm)^a^	7.3 ± 0.1	7.4 ± 0.3
Stem diameter (mm)^b^	7.6 ± 0.1	8.7 ± 0.1*
Number of lateral shoots^a^	0.6 ± 0.2	0.0 ± 0.0*
Number of lateral shoots^b^	2.2 ± 0.2	0.5 ± 0.1*
5^th^ internode length (mm)	20.0 ± 1.0	26.0 ± 1.0*
**Reproductive traits**		
Number of flowers	14.2 ± 0.5	10.3 ± 0.8*
Fruit set (%)	80.7 ± 1.3	82.6 ± 3.8
Number of petals^c^	5.1 ± 0.0	5.3 ± 0.1*
Number of sepals^c^	5.2 ± 0.1	5.7 ± 0.1*
Days to first anthesis (DAS)	34.0 ± 0.3	40.0 ± 0.5*
Days to first breaker (DAS)	76.0 ± 0.6	80.0 ± 1.0*
Fruit development (days)^d^	41.7 ± 0.4	39.3 ± 0.7*
**Fruit-related traits**		
Fruit weight (g)^e^	5.1 ± 0.2	4.9 ± 0.2
Fruit diameter (mm)^e^	22.2 ± 0.4	21.7 ± 0.3
Fruit shape index^e^	0.9 ± 0.1	0.9 ± 0.1
Pericarp thickness (mm)^e^	2.1 ± 0.0	2.5 ± 0.0*
Number of locules/fruit^e^	2.8 ± 0.1	2.8 ± 0.1
Number of seeds/fruit^e^	49.6 ± 2.0	28.7 ± 1.5*
Total soluble solids (Brix)^e^	4.5 ± 0.1	5.6 ± 0.1*
Fruit firmness (gf)^f^	324.5 ± 9.1	326.4 ± 10.5
Fruit brightness (L*)^f^	48.8 ± 0.2	47.7 ± 0.4*
a*/b*^f^	0.8 ± 0.0	1.0 ± 0.0*

Values are mean SE (n14). Mean values of slhws-1 followed by asterisk (*) are significantly different from the mean value of the WT according to a t-student test P < 0.05. DAS, days after sowing. ^a^Measured on 30 DAS. ^b^Measured on 60 DAS. ^c^Average of 50 flowers. ^d^Calculated from the number of days between the anthesis and breaker stages. ^e^Average of 50 fruits. ^f^Average of 20 fruits.

### *Slhws-1* and *Slhws-2* Show Flowering Delay, High Brix and High Estimated Lycopene Content

Flowering time was measured as the number of days from sowing to the anthesis of the first flower. The *slhws-1* and *slhws-2* mutants showed a three to six-day delay compared to the WT depending on the culture conditions (**Table 4**; **Supplementary Table S6**). Interestingly, the time for fruit development, measured as the number of days from anthesis to breaker stage, was shorter of about two days in the *slhws-1* mutant (**Table 4**). Fruit quality-related traits were differently affected between *slhws-1* and *slhws-2* and depending on the culture conditions, however, total soluble solids (°Brix) and lycopene content estimation (a*/b* ratio) were consistently higher in the mutants (**Table 4**; **Supplementary Table S6**). Both *slhws-1* and *slhws-2* also showed significantly thicker pericarp, a feature commonly observed in other parthenocarpic mutants (De Jong et al., 2009; Carrera et al., 2012; Ding et al., 2013; Okabe et al., 2018).

### *Slhws* Expression Levels in *Slhws-1*

**Figure 9:**
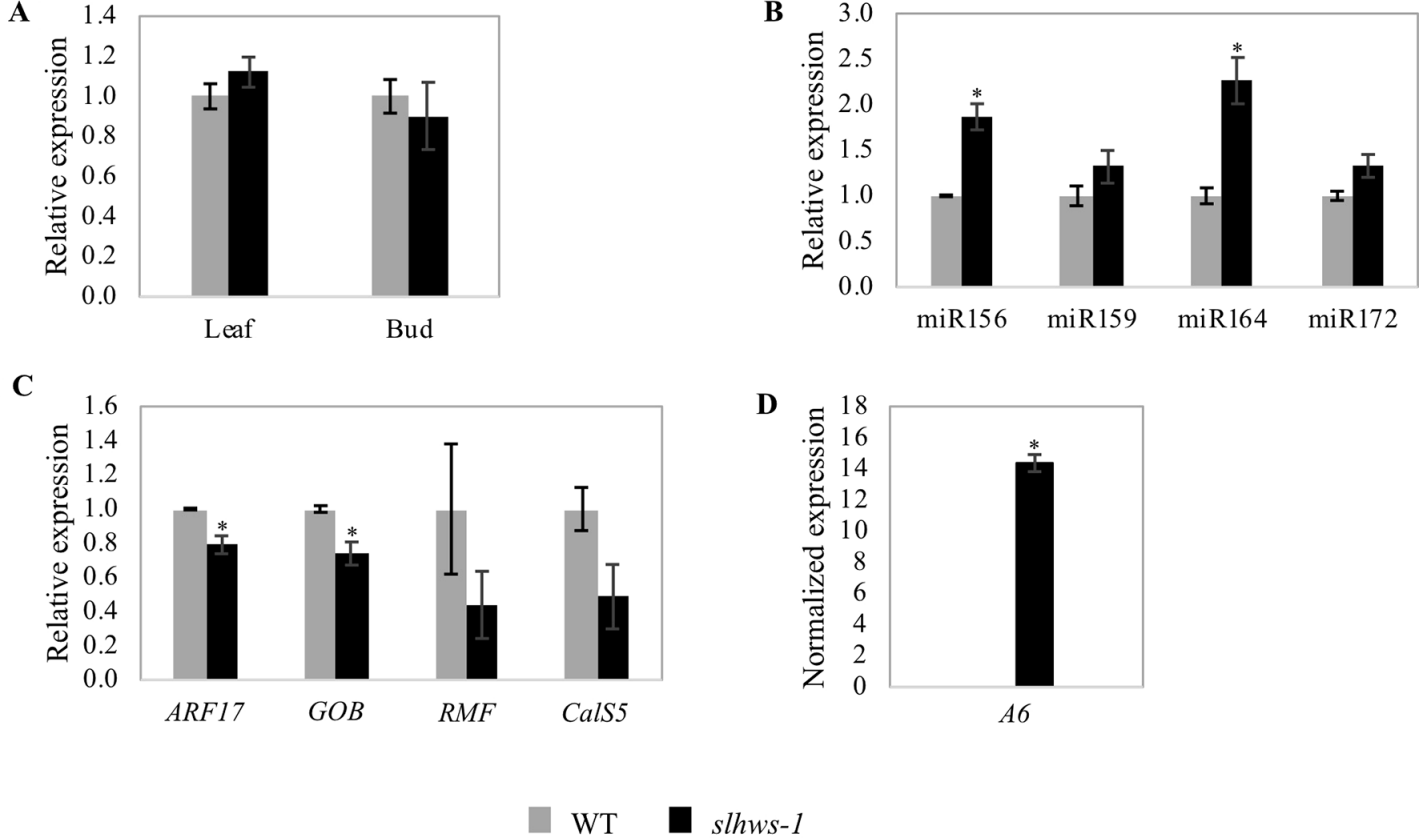
Expression levels of miRNAs and genes of interest in *slhws-1* measured by RT-qPCR. **(A)** Relative *SlHWS* expression in leaf and bud tissues of WT and *slhws-1*. **(B)** Relative expression of miRNAs of interest in WT and *slhws-1* buds. **(C)** Relative expression of *ARF17*, *GOB*, *RMF* and *CalS5* in WT and *slhws-1* buds. **(D)** Normalized expression of *A6* in WT and *slhws-1* buds. Gene expressions were normalized using the *SAND* gene as reference. Values are mean SE. Mean was calculated from three biological replicates and each biological replicate was analyzed in three technical replications. *, significantly different from the mean value of the WT according to a t-student test *P* < 0.05.

In *slhws-1*, the mutation results in an amino acid change which is believed to alter the function of HWS within the SCF protein complex. To confirm that no feedback regulation was occurring in the mutant, mRNA levels were investigated in leaf and bud tissues by RT-qPCR. In both tissues, gene expression levels were comparable to the ones of the WT, suggesting that partial loss of function of *HWS* does not induce any feedback regulation mechanisms (**Figure 9A**).

### Changes in the Expression Level of Genes and MicroRNAs Related to the *Slhws-1* Phenotype

Several studies have described the role of microRNAs (miRNAs) in plant development (D’Ario et al., 2017; Djami-Tchatchou et al., 2017; Liu et al., 2018). Relevant to the phenotype of *slhws-1*, miR156 and miR172 primarily control flowering time (Spanudakis and Jackson, 2014; Teotia and Tang, 2015); miR159 is involved in fruit development in tomato (da Silva et al., 2017) and altered levels of miR164 have been linked to the reduction in leaf serration in *athws-1*, most likely via the downregulation of the *CUP-SHAPED COTYLEDON 1* (*CUC1*) and *CUC2* transcription factors (Lang et al., 2018). Levels of miR156, miR172, miR159, and miR164 were thus investigated. A sharp increase in the level of miR156 was measured in the mutant, in accordance with the delayed flowering time observed earlier (**Figure 9**, **Table 4**). In contrast to the action of miR156, miR172 has been shown to promote flowering and the transition to adult phase. A non-significant increase in levels of miR172 was measured in *slhws-1*, suggesting that the flowering delay phenotype was mainly influenced by action of miR156. Parthenocarpy can be induced by overexpressing miR159 (da Silva et al., 2017). For this miRNA also a non-significant increase was measured in the mutant. In contrast, miR164 was found to be significantly increased in *slhws-1* buds (**Figure 9**). The tomato ortholog of *CUC1* and *CUC2* is the *GOBLET* (*GOB, Solyc07g062840*) gene. Overexpression of miR164 was shown to result in reduction in tomato leaf serration via *GOB* repression (Berger et al., 2009). It was found that levels of miR164 were more than doubled in *slhws-1* while *GOB* mRNA levels were decreased in a RT-qPCR experiment (**Figure 8**), in accordance with results from Berger et al. (2009) and Lang et al. (2018) studies respectively. In *A. thaliana*, the *REDUCED MALE FERTILITY* (*RMF*) gene is involved in tapetum degeneration during anther development (Kim et al., 2010). The dominant mutant *rmf-1D* showed vacuolated tapetum and low male fertility phenotypes. The tomato putative ortholog of *RMF, Solyc01g095870,* was found to be decreased in *slhws-1* buds, however the decrease was not statistically significant, possibly because the whole buds and not stamens were used in the experiment (**Figure 9**). Downregulation of AUXIN RESPONSE FACTOR 17 (*ARF17*) results in pollen development defects and associated male sterility in *A. thaliana* (Yang et al., 2013a). *CALLOSE SYNTHASE5* (*CalS5*) is a major gene for callose biosynthesis and is downregulated in the *arf17* mutant. In our study, *SlARF17* (*Solyc11g013480*) was found to be significantly decreased by RT-qPCR (**Figure 9**), suggesting that the low pollen viability in *slhws-1* results, at least partially, from the low abundance of ARF17. *SlCalS5* (*Solyc11g005980*) expression level was also decreased in the mutant, however not to statistically different levels according to a Student’s t test. In pollen sacs, callose is degraded by a callase enzyme encoded by the *A6* (*Solyc12g098560*) gene. At stage 9 of flower development, *A6* expression was not detected in the WT. In contrast, the gene was markedly expressed in the mutant, strongly suggesting that early callose degradation contributes to the pollen development defects in *slhws-1* (**Figure 9D**).

## Discussion

In this study, we report the isolation of *slhws-1*, a tomato mutant showing facultative parthenocarpy, leaflet fusion, reduction in leaflet serration and reduced fertility. The F-box encoding gene *Solyc01g095370* was found to carry an amino acid change mutation in *slhws-1* (**Figure 2**). Two additional alleles, namely *slhws-2* and *slhws-3*, were identified via TILLING and confirmed in allelism tests. All *slhws-1, slhws-2* and *slhws-3* lines showed the fused-leaflet, reduction in leaflet serration (**Figure 1**; **Supplementary Figure S4**) and parthenocarpy phenotypes (**Table 2**). Examination of key phenotypic traits were found to be consistent between *slhws-1* and *slhws-2*. Furthermore, the *slhws-1* allele was introduced into the cv. Aichi First and cv. Ueleie 106 WP genetic backgrounds and NILs BC_2_ individuals exhibited leaflet fusion, reduction in leaf serration and seedless fruits, providing additional data for the causal role of *slhws-1* in these phenotypes (**Supplementary Figure S5**).

Database analysis identified *AtHWS* as the putative ortholog of *SlHWS*, with respective proteins showing more than 60% homology (**Figure 2C**). The *hws* mutant was first described for its fused floral organ phenotype and fused sepals along the lower part of their margin. Occasional filament fusions as well as filament-to-silique fusions have also been reported (González-Carranza et al., 2007). Histological sections of *slhws-1* showed strikingly similar phenotypes (**Figure 8**). Recent studies in *A. thaliana* revealed a role for *HWS* in miRNA function (Zhang et al., 2017; Lang et al., 2018). The *slhws-1* showed about a two-fold increase in miR164 levels and lower *GOB* transcript accumulation (**Figures 9B, C**). In the *athws-1* mutant, partial cauline leaf fusion to the inflorescence and reduction in leaf serration are described as a result of miR164 transcripts accumulation (Lang et al., 2018). miR164 cleaves *CUC1* and *CUC2* mRNAs, resulting in reduction in leaf margin serration (Nikovics et al., 2006). Rosette leaf fusion and cauline leaf fusion to the inflorescence have also been observed in miR164b overexpressor lines of *A. thaliana* (Mallory et al., 2004). *GOB*, the tomato ortholog of *CUC*, is also targeted by miR164 and the *gob-3* mutant exhibits a fused leaflet phenotype (Berger et al., 2009) that is resembling the one observed in our mutant. Additionally, *slhws-1* and *slhws-2* showed arrested apical meristem development and produced a cup-shaped-like first leaf (**Supplementary Figures S2A, B**), which is similar to the cup-shaped cotyledon observed in the *cuc1cuc2* double mutant of *A. thaliana* (Aida et al., 1997) and to the tomato *gob-3* mutant (Berger et al., 2009). Similar phenotypes were additionally shown by NILs of *slhws-1* in both cv. Aichi First and cv. Ueleie 106 WP genetic backgrounds (**Supplementary Figures S2C, D**).

In addition to its function in flower development (González-Carranza et al., 2007; González-Carranza et al., 2017), *HWS* has been also shown to be involved in root development and root meristem activity in poplar and *A. thaliana*, respectively (Dash et al., 2015; Kim et al., 2016), as well as in photosynthesis activity in rice (Yu et al., 2015). However, there are no reports about the function of *HWS* in fruit development. The *slhws-1* is able to produce seeds, although the amount of seed produced is largely influenced by the growth conditions (**Table 4**; **Supplementary Table S6**). In high temperature and humidity conditions, seed production is lower and parthenocarpy is favored. High temperatures have been shown to induce parthenocarpy (Sato et al., 2001) and high humidity is known to hinder dehiscence (Gradziel and Weinbaum, 1999). Partial recovery of *slhws-1* mutant seed formation was observed during autumn cultivation (**Figure 1**; **Tables 2** and **4**), when average temperature and humidity were low, while severely reduced seed production was observed during high temperature cultivation (**Supplementary Figure S1**). A similar pattern was observed on emasculated flowers, with more parthenocarpic fruits development in summer cultivation (**Table 2**). Partial restoration of seed production ability in low temperature has also been reported in stamenless mutants (*sl* and *sl2* mutants) (Sawhney, 1983; Polowick and Sawhney, 1995; Gómez et al., 1999) and in the *pat* mutant (Mazzucato et al., 1998).

It has been shown that the low seed number in *slhws-1* is mainly due to a defective male organ function (**Figures 8**). Reduction in the number of pollen mother cells (PMCs) and megaspore mother cells (MMCs) has been reported in loss-of-function mutants of the miRNA biogenesis pathway such as *hyponastic leaves1 (hyl1)*, *hua enhancer1 (hen1)*, *dicer-like1 (dcl1)*, *hasty (hst)*, and *argonaute1 (ago1)*, which all show reduced fertility (Oliver et al., 2017). Furthermore, miR164 has been shown to be involved in the establishment and maintenance of gynoecium development, notably via the regulation of the *GOB* gene (Correa et al., 2018). In *slhws-1*, miR164 levels are higher and *GOB* levels are lower, suggesting that the female function might also be affected in the mutant (**Figures 9C, B**, respectively). Yet, no striking female organ defects could be observed during our histological analysis and seed number was not significantly different in the mutant when WT pollen was used for pollination (**Figure 7**). Presumably, the change of expression in miR164 and *GOB* in the mutant is not large enough to induce gynoecium developmental defects and the female function remains unaffected.

Defects in anther and tapetum developments and their impact on male fertility have been documented in a number of mutants in *A. thaliana* (Zhang et al., 2006; Ito et al., 2007; Yang et al., 2007; Zhu et al., 2008; Kim et al., 2010; Gu et al., 2014), rice (Jung et al., 2005; Li et al., 2006), tomato with the *male sterile 10* *^35^* (*ms10* *^35^*) and *7B-1 male-sterile* mutants (Jeong et al., 2014; Omidvar et al., 2017), and natural variants in kiwifruit (Falasca et al., 2013) and orchid (Hu et al., 2018). In *slhws-1*, reduction in pollen number and lower pollen viability was associated with enlarged and vacuolated tapetal cells (**Figures 8A, D**) similar to the ones described in the *ms10* *^35^* and the *rmf-1D* mutants (Kim et al., 2010; Jeong et al., 2014). Expression of *RMF* was found to be decreased in an RT-qPCR experiment, however not significantly (**Figure 9**). Investigation of the level of expression of this gene in the anther tissue should allow confirming the decrease in *slhws*.

During microsporogenesis, callose functions as a temporary cell wall that prevents the microspores from fusing together (Scott et al., 2004). Expression of the *CalS5* gene, which encodes an enzyme involved in callose, is regulated by *ARF17*. In the *arf17* mutant, *CalS5* levels are lower, resulting in pollen wall patterning defects and pollen degradation (Yang et al., 2013a). The putative ortholog of *ARF17* was found to be markedly downregulated in *slhws-1* (**Figure 9**). Reduction in *ARF17* is expected to result in lower *CalS5* transcript accumulation in *slhws-1* and in lower pollen germination (**Figure 6**) as both genes are required for pollen tube growth (Dong et al., 2005; Yang et al., 2013a; Shi et al., 2015). However, *CalS5* was found to be slightly reduced but not significantly different from the WT (**Figure 9**). *ARF17* has been reported to be cleaved by miR160 (Mallory et al., 2005). miR160 has not been quantified in *slhws*-1, however Mei et al. (2019) have reported that the level of miR160 are unchanged in *athws-6* loss-of-function mutant. It was hypothesized that *HWS* may only affect a subset of miRNAs, accounting for the unchanged levels in miR160 (Mei et al., 2019). After microsporogenesis, the callose wall is degraded by β-1,3-glucanase (callase) produced by the tapetum to release microspores from the tetrads (Scott et al., 2004). Premature dissolution of the microspore callose wall has been reported to cause male sterility in tobacco (Worrall et al., 2004). Callase is predominantly synthesized from the *A6* gene, which has been reported to be positively regulated by *MYB80* in *A. thaliana* (Hird et al., 1993; Zhang et al., 2007). In *slhws-1* stage 9 buds, *A6* was markedly expressed, while no expression could be detected in WT counterparts. This suggests that the callase enzyme is prematurely active in the mutant, leading to pollen development defects.

miR156 and miR172 have been extensively studied for their role in regulating flowering time. miR156 is strongly expressed during early shoot development and miR156 levels gradually decline as the plant grows (Yang et al., 2010). Overexpression of SlymiR156a has been shown to result in flowering delay in tomato (Zhang et al., 2011). On the other hand, miR172 levels gradually increase toward flowering stages, accumulating in leaves and floral buds (Aukerman and Sakai, 2003; Wu et al., 2009). Analyses of tomato overexpressor lines of miR164 revealed that the duration of specific fruit developmental stages was influenced by miR164 levels (Rosas Cárdenas et al., 2017). In our experiments, *slhws-1* showed significant increase in miR156 and miR164 levels (**Figure 9**), which are comparable for the most part to the ones reported in previous *athws-1* mutant studies (Zhang et al., 2017; Lang et al., 2018). The mutant showed a flowering delay of about six days (**Table 4**), which is presumably resulting from the significantly increased miR156 levels (**Figure 9**). Higher levels of miR156 are expected to result in a lower or delayed expression of miR172 via repression of some *SPL* transcription factor genes (Spanudakis and Jackson, 2014). Those two miRNAs act antagonistically in determining the vegetative to flowering transition (Teotia and Tang, 2015). In *hws* mutants, the degradation mechanism of miRNAs is impaired, leading to an accumulation of all synthesized miRNA species (Lang et al., 2018; Mei et al., 2019). It is not clear how the defect in miRNA degradation interferes in the balance between miR156 and miR172, still the sharp difference in miR156 levels observed in *slhws-1*, in contrast to the small change in miR172 levels, suggests that miR156 action is dominant in the determining the phenotype. This idea is supported by the flowering delay observed, characteristic of elevated miR156 levels. Maintained abnormal levels of miR164 are most likely responsible for shorter anthesis-to-fruit development time (**Table 4**). Based on the above results, it is hypothesized that the parthenocarpy phenotype in *slhws-1* results from the deregulation of the fruit development timing via elevated levels of key miRNAs. Developmental stage-specific and tissue-specific sampling is needed to study more precisely the modulation of those miRNAs and their target in *slhws-1*.

da Silva et al. (2017) showed that parthenocarpic fruits can be obtained in tomato by overexpressing miR159 and that this overexpression was associated with a decrease in levels of gibberellin-regulated GAMYB transcription factors. In the buds of *slhws-1*, miR159 was found to be only slightly increased compared to the WT, with no statistical difference (**Figure 9**). Analysis of the expression pattern of miR159 showed that expression levels are relatively low in buds compared to pre-anthesis ovaries (da Silva et al., 2017). For this reason, analysis of miR159 at a later stage, i.e. around anthesis, is expected to reveal a more pronounced expression difference between *slhws-1* and the WT.

Most fruit quality-related traits were not significantly different when comparing *slhws-1* seeded fruits and WT fruits. Higher soluble solids content (Brix), which is used to approximate sugar content, and a*/b* ratio, which is reflective of the lycopene content (Arias et al., 2000), were both higher in the mutant (**Table 4**; **Supplementary Table S6**). Increase in Brix value has been reported in some parthenocarpic tomato lines (Carmi et al., 2003; Medina et al., 2013). Higher Brix index and lycopene content have also been reported in the *procera-2* parthenocarpic mutant, a novel allele of *SlDELLA* (Shinozaki et al., 2018).

The leaflet fusion and reduction in leaflet serration phenotypes in *slhws-1* are similar to the ones described in auxin distribution mutants. Leaflet fusion was described in parthenocarpic *sliaa9*-silenced plants (Wang et al., 2005) and *Aucsia*-silenced plants (Molesini et al., 2009). Significant reduction in the number of lateral shoots suggested that auxin transport was defective in *slhws-1* since polar auxin transport is required for lateral shoot formation (Wang et al. 2014). Several F-box proteins have been reported to be involved in hormonal response and signaling (Potuschak et al., 2003; Kepinski and Leyser, 2005; Yu et al., 2007; Ariizumi et al., 2010; Hu et al., 2012), and miRNAs have been reported as key regulators in hormonal response pathways (Curaba et al., 2014). Altogether, these data suggest a role for *SlHWS* in hormone-driven morphological development via modulation of the miRNA biogenesis pathway.

In summary, this study is the first description of *SlHWS*, the putative ortholog of the *A. thaliana HAWAIIAN SKIRT* gene. The *slhws-1* mutant showed phenotypes that are resembling the ones observed in the *athws-1* mutant. This study describes a novel function for *HWS* in fruit development based on the facultative parthenocarpy phenotype in tomato. Parthenocarpic fruit formation in *slhws-1* was associated with anther developmental and flower morphological alterations, resulting in reduced fertility. The reduction in seed formation was partially recovered when the mutant was grown in favorable temperature conditions. The *slhws-1* showed changes in some miRNA levels, notably elevated miR164 levels, which are hypothesized to be responsible for the observed leaf, flower and fruit development phenotypes. In future studies, it would be interesting to investigate the expression levels of relevant miRNAs at specific developmental stages and at tissue level to further dissect the function of *SlHWS* in miRNA biogenesis, anther development and hormonal regulation.

## Data Availability Statement

All datasets generated for this study are included in the manuscript/**Supplementary files**.

## Author Contributions

YO, NW, TA, and NF contributed to the mutant screening. FD, J-IM, TI, KH, YO, and FL performed phenotypic characterizations of mutants. J-IM, FD, NW, and TA contributed to genetic mapping and gene identification. FL, FD, and YS performed quantitative measurements of transcripts including miRNAs. FD, FL, and HE wrote the manuscript. All authors reviewed and approved the final manuscript.

## Funding

This work was supported by JSPS KAKENHI, Grant No. 25252008 and Grant No. 17H01461 to HE, and Science and Technology Research Promotion Program for Agriculture, Forestry, Fisheries and Food Industry, Japan (Grant No. 26013A) to HE.

## Conflict of Interest

The authors declare that the research was conducted in the absence of any commercial or financial relationships that could be construed as a potential conflict of interest.
