# Supplementary figures and images for: Functional Disruption of the Tomato Putative Ortholog of HAWAIIAN SKIRT Results in Facultative Parthenocarpy, Reduced Fertility and Leaf Morphological Defects
Source: Front Plant Sci. 2019 Oct 14;10:1234. doi: 10.3389/fpls.2019.01234 (PMC6801985; doi:10.3389/fpls.2019.01234)

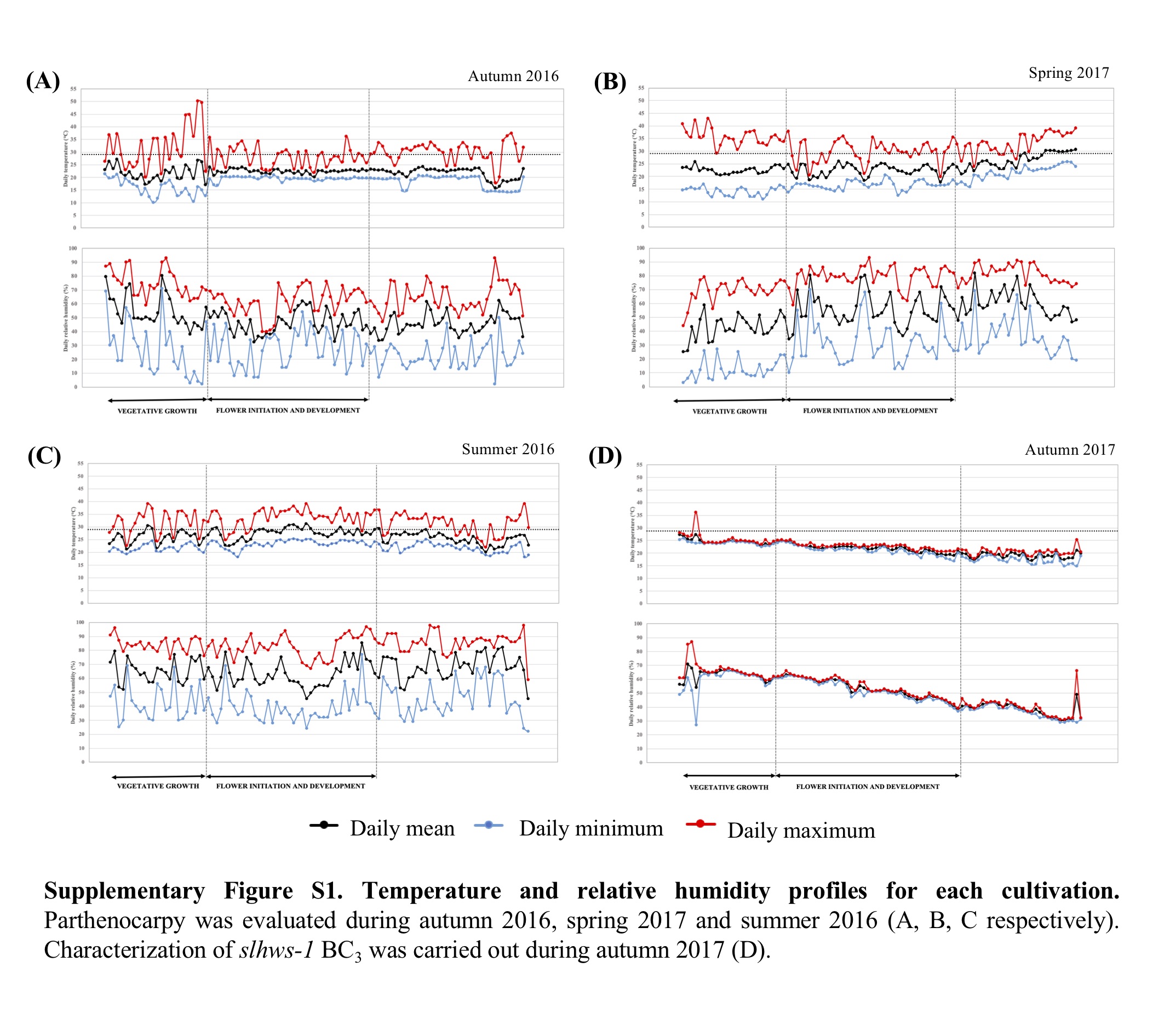

Supplement: Supplementary file 1 [file Image_1.jpeg]

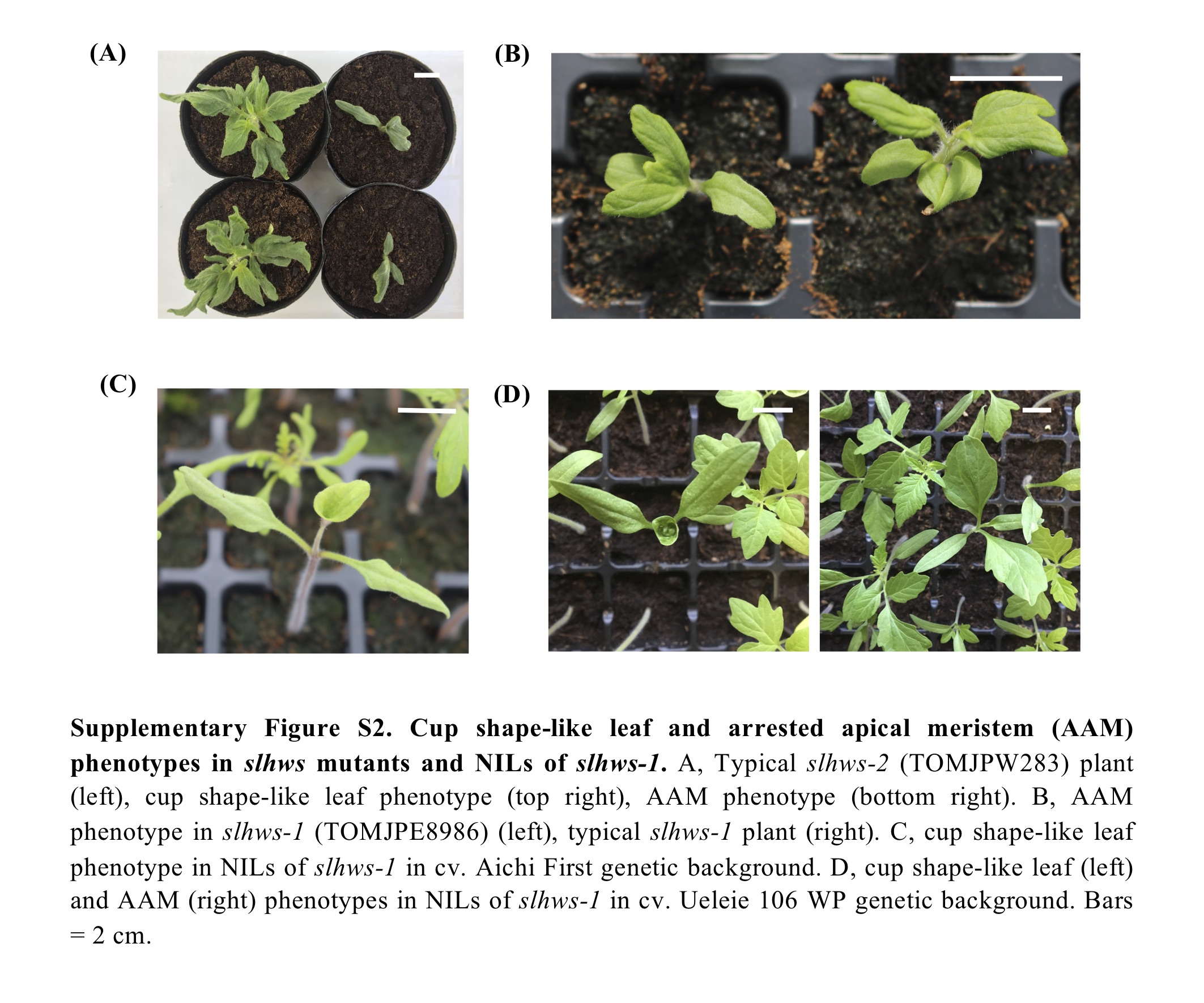

Supplement: Supplementary file 2 [file Image_2.jpeg]

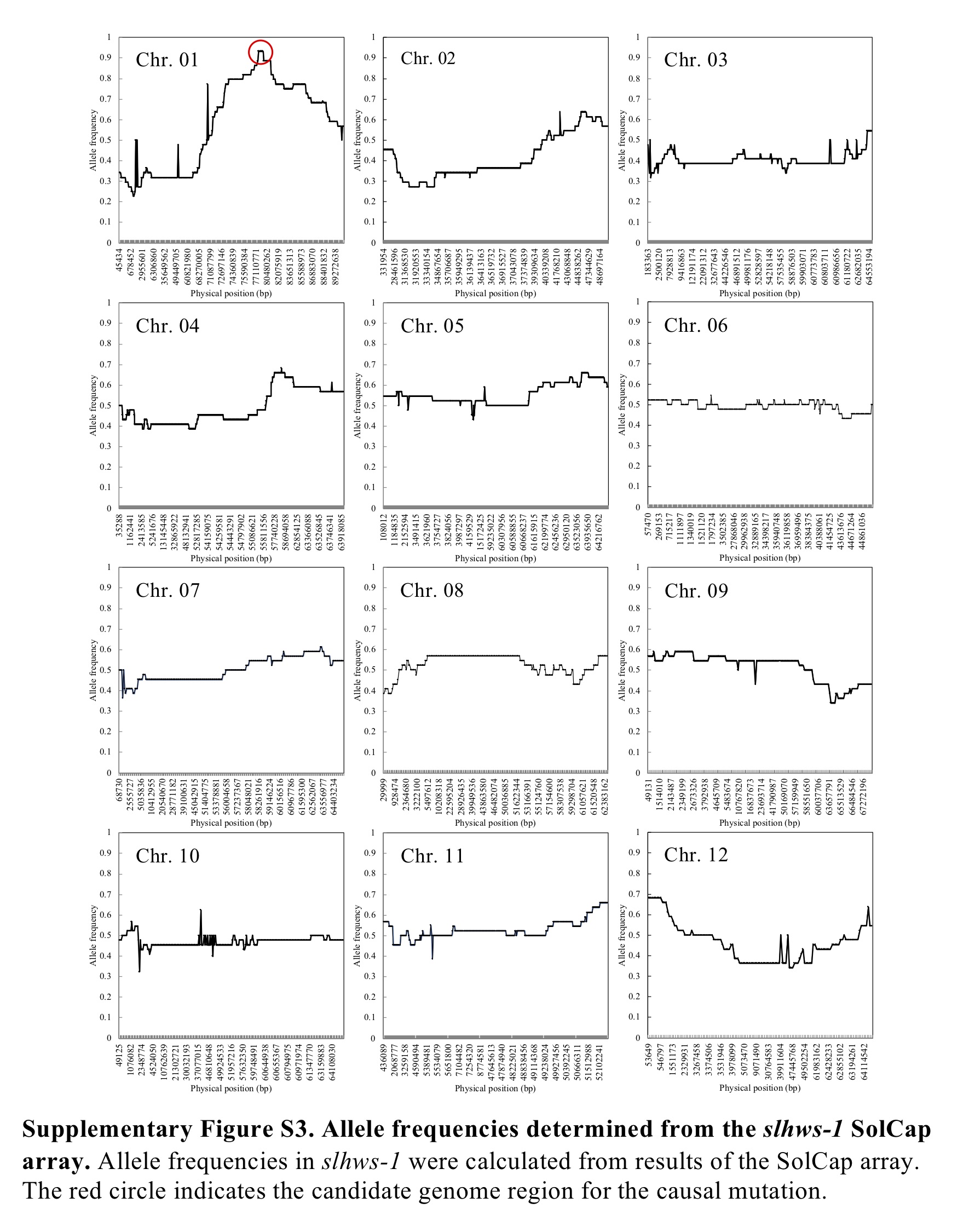

Supplement: Supplementary file 3 [file Image_3.jpeg]

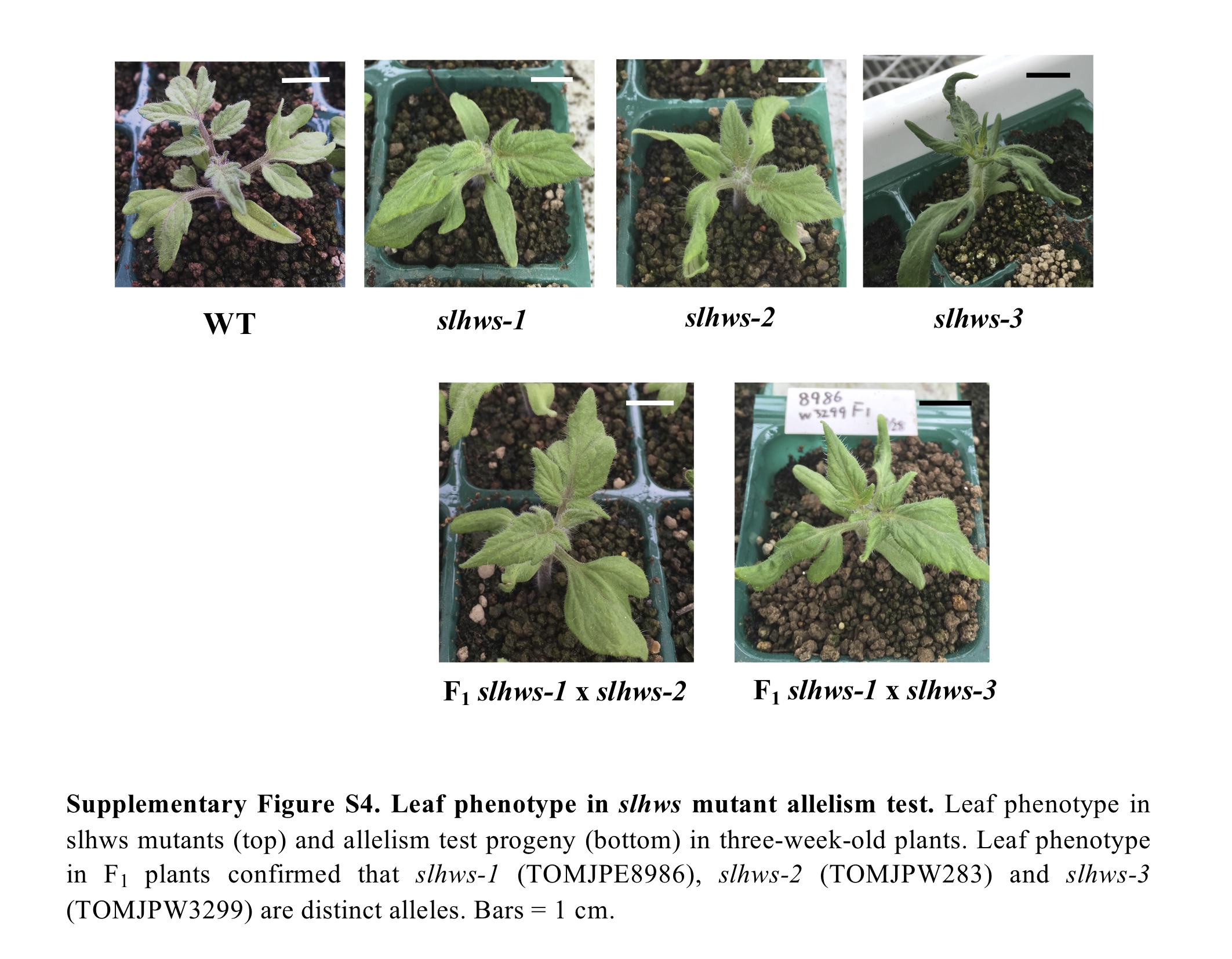

Supplement: Supplementary file 4 [file Image_4.jpeg]

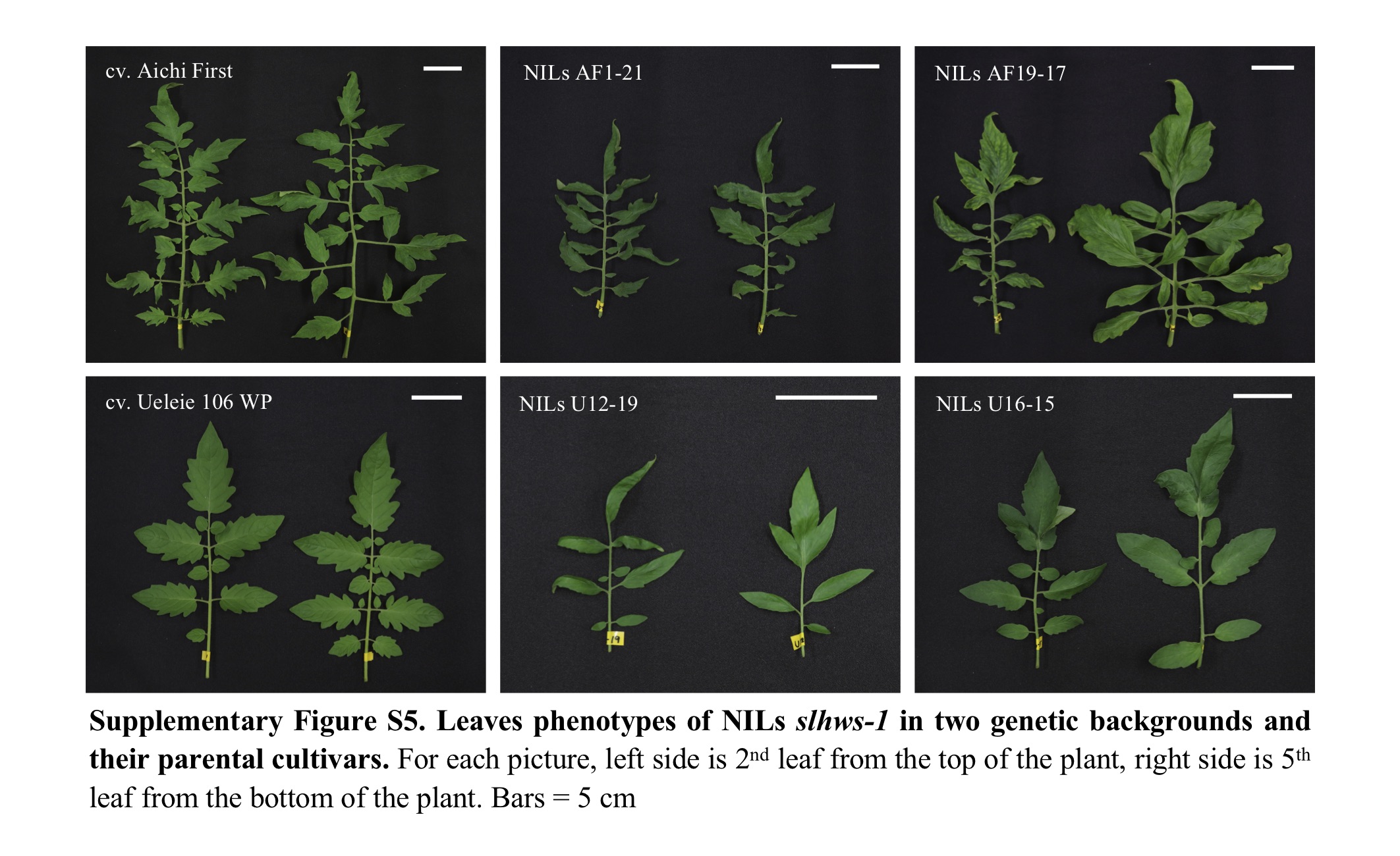

Supplement: Supplementary file 5 [file Image_5.jpeg]
