# Supplementary material for: Functional Disruption of the Tomato Putative Ortholog of HAWAIIAN SKIRT Results in Facultative Parthenocarpy, Reduced Fertility and Leaf Morphological Defects
Source: Front Plant Sci. 2019 Oct 14;10:1234. doi: 10.3389/fpls.2019.01234 (PMC6801985; doi:10.3389/fpls.2019.01234)
Supplement: Supplementary file 6 [file Table_1.docx]

**Supplementary Tables**

Supplementary Table S1. Primers used in this study.

| ***dCAPS marker primers*** | | |
| --- | --- | --- |
| **Primer name** | **Forward primer sequence (5’-3’)** | **Reverse primer sequence (5’-3’)** |
| E8986-dcaps  (*Solyc01g095370*) | CCCGCATGCCACACAAGTATTT | ATCACATATCAGGGAGACATCTCAAGCCAT |
| ***RT-qPCR primers*** | | |
| **Primer name** | **Forward primer sequence (5’-3’)** | **Reverse primer sequence (5’-3’)** |
| miR156-RT | CTTCTGTCAACTATGCTCTCCA | GCCTTGACAGAAGATAGAGAGC |
| miR159-RT | TCAATCCAAATCATGTTCTCC | TTTGGATTGAAGGGAGCTCTA |
| miR164-RT | TGCTTCTCCACTATCCTCTCC | TGGAGAAGCAGGGCACGT |
| miR172-RT | TCAAGATTCTCTATACTCTCCAGG | AGAATCTTGATGATGCTGCATAAG |
| SlHWS-RT  (*Solyc01g095370*) | GGTTATGCCTATGATCCTTCCC | TCGCTTCTACTGTCATTGTCC |
| ARF17-RT  (*Solyc11g013480*) | TTATCTGTTCGTTTTCTCGC | TCCCTCATAGTAACTTCATC |
| GOB-RT  (*Solyc07g062840*) | AATATCGCCTTGATGGCAAA | GAGAGACGGAAGACGGTGAA |
| RMF-RT  (*Solyc01g095870*) | CTAGGTTCCCACAACGAGAAG | TTTCCTACCCATGACTTGAGATC |
| CalS5-RT  (*Solyc11g005980*) | GTCATGCTGTTCAAATTCCTAAGTC | TTCACAGATCCCCACATTCC |
| A6 (*Solyc12g098560*) | GCCAAGTATAGGGAAGATGGTG | CACTAAAGAGAAACATATGGAAATTTGC |
| SAND-RT  (*Solyc03g115810*) | TTGCTTGGAGGAACAGACG | GCAAACAGAACCCCTGAATC |
| ***two-tailed RT-qPCR* *primers*** | | |
| miR156-tt | CTTCTGTCAACTATGCTCTCCAGACACAGTTGGTGTCTGTCTCCACTTGTGCTC | |
| miR159-tt | TCAATCCAAATCATGTTCTCCAGGTACAGTTGGTACCTGTGTCCACTTTAGAG | |
| miR164-tt | TGCTTCTCCACTATCCTCTCCAGGTACAGTTGGTACCTGTCTCCACTTTGCAC | |
| miR172-tt | TCAAGATTCTCTATACTCTCCAGGTACAGTTGGTACCTGTCTCCACTTATGCA | |
| ***TILLING primers*** | | |
| **Primer name** | **Forward primer sequence (5’-3’)** | **Reverse primer sequence (5’-3’)** |
| F-box-TILL1-2 | CCACAGCCTTAGGGTTGGAG | ACGGTCTGGTTTCCCAATCC |
| F-box-TILL2-2 | GCATTGGCTATCTGTGCAAACA | AGACAACTTGTCCCTGTCTTGG |

Supplementary Table S2. Genetic analysis of mutant phenotype.

| Cross | Generation | Number of plants with associated phenotype | | χ2 value | | *P* value | |
| --- | --- | --- | --- | --- | --- | --- | --- |
|  |  | Normal leaf morphology  (WT-like) | Abnormal leaf morphology  (Mutant-like) | |  | |  |
| WT x *slhws-1* | F_1_ | 6 | 0 | | - | | - |
|  | F_2_ | 77 | 30 | | 0.53 | | 0.47 |

Inheritance pattern was determined based on the χ2 value. Significant difference was estimated at level of 0.05.

Supplementary Table S4. Phenotype and genotype observed in F_2_ generation.

| Cross combination | Observed phenotypes in F_2_ population | | | | | | | Expected ratio (W/W:W/m:m/m) | | χ2 value | | *P* value | |  |
| --- | --- | --- | --- | --- | --- | --- | --- | --- | --- | --- | --- | --- | --- | --- |
|  | Wild type-like | | | Mutant-like | | | |  |  |  |  |  |  |  |
|  | Genotype | | | Genotype | | | |  |  |  |  |  |  |  |
|  | W/W | W/m | m/m | | W/W | W/m | m/m | |  | |  | |  | |
| WT x *slhws-1* | 38 | 66 | 0 | | 0 | 0 | 24 | | 1:2:1 | | 3.19 | | 0.2 | |

Inheritance pattern was determined based on the χ2 value. Significant difference was estimated at level of 0.05.

Abbreviations: W/W, Homozygous dominant for the wild type allele; W/m, Heterozygote; m/m, Homozygous recessive for the mutant allele.

Supplementary Table S5. Allelism test of *slhws-1* x *slhws-3*.

| Cross | Generation | Number of plants with associated phenotype | | | χ2 value | *P* value |
| --- | --- | --- | --- | --- | --- | --- |
|  |  | Normal leaf morphology  (WT-like) | Abnormal leaf morphology  (Mutant-like) |  | |  |
| Homozygous *slhws-1* x  Heterozygous *slhws-3* | F_1_ | 3 | 4 | 0.14 | | 0.71 |

Inheritance pattern was determined based on the χ2 value. Significant difference was estimated at level of 0.05.

Supplementary Table S6. Characterization of *slhws-1* and *slhws-2* in spring season.

| Traits | WT | | *slhws-1* | | *slhws-2* | |  |
| --- | --- | --- | --- | --- | --- | --- | --- |
| **General architecture** | |  | |  | |  | |
| Plant height (cm) ^a^ | | 8.3 + 0.2 | | 6.8 + 0.2 * | | 3.6 + 0.2 * | |
| Stem diameter (mm) ^a^ | | 6.0 + 0.2 | | 5.1 + 0.2 * | | 4.1 + 0.3 * | |
| Number of lateral shoots ^a^ | | 3.6 + 0.1 | | 0.2 + 0.0 * | | 0.5 + 0.2 * | |
| **Reproductive traits** | |  | |  | |  | |
| Number of flowers | | 12.8 + 0.8 | | 6.9 + 0.4 * | | 6.4 + 0.4 * | |
| Fruit set (%) | | 90.0 + 2.4 | | 85.0 + 3.0 | | 18.2 + 6.2 * | |
| Days to first anthesis (DAS) | | 35.0 + 0.3 | | 39.0 + 0.2 * | | 38.0 + 0.3 * | |
| **Fruit related traits** | |  | |  | |  | |
| Fruit weight (g) ^b^ | | 9.2 + 0.4 | | 8.1 + 0.3 * | | 5.2 + 0.4 * | |
| Fruit diameter (mm) ^b^ | | 28.2 + 0.3 | | 26.0 + 0.2 * | | 20.8 + 0.5 * | |
| Fruit shape index ^b^ | | 0.8 + 0.0 | | 0.9 + 0.0 * | | 1.0 + 0.0 * | |
| Pericarp thickness (mm) ^b^ | | 2.6 + 0.1 | | 3.4 + 0.1 * | | 3.7 + 0.1 * | |
| Number of locules/fruit ^b^ | | 3.7 + 0.2 | | 3.8 + 0.1 | | 3.1 + 0.1 * | |
| Number of seeds/fruit ^b^ | | 60.0 + 3.6 | | 0.1 + 0.1 * | | 0.3 + 0.1 * | |
| Total soluble solids ($^{\circ}$Brix) ^b^ | | 4.4 + 0.1 | | 6.3 + 0.1 * | | 5.9 + 0.1 * | |
| Fruit firmness (gf) ^c^ | | 436.3 + 10.6 | | 437.4 + 9.1 | | 475.0 + 20.7 | |
| Fruit brightness (L*) ^c^ | | 46.5 + 0.3 | | 43.2 + 0.8 * | | 38.7 + 0.5 * | |
| a*/b* ^c^ | | 0.8 + 0.0 | | 1.0 + 0.0 * | | 1.1 + 0.0 * | |

Values are means + SE (n>11plants). Mean values of *slhws-1* and *slhws-2* followed by asterisk (*) are significantly different from the mean value of the WT according to a t-student test *P* <0.05. *DAS,* days after sowing. ^a^, measured on 30 DAS. ^b^, average of 50 fruits. ^c^, average of at least 5 fruits.
